# More Than Just Attractive: How CCL2 Influences Myeloid Cell Behavior Beyond Chemotaxis

**DOI:** 10.3389/fimmu.2019.02759

**Published:** 2019-12-13

**Authors:** Martha Gschwandtner, Rupert Derler, Kim S. Midwood

**Affiliations:** ^1^Kennedy Institute of Rheumatology, Nuffield Department of Orthopaedics, Rheumatology and Musculoskeletal Sciences, University of Oxford, Oxford, United Kingdom; ^2^Department of Pharmaceutical Chemistry, Institute of Pharmaceutical Sciences, University of Graz, Graz, Austria

**Keywords:** chemokine, CCL2, macrophages, myeloid cells, polarization, function, immune oncology

## Abstract

Monocyte chemoattractant protein-1 (MCP-1/CCL2) is renowned for its ability to drive the chemotaxis of myeloid and lymphoid cells. It orchestrates the migration of these cell types both during physiological immune defense and in pathological circumstances, such as autoimmune diseases including rheumatoid arthritis and multiple sclerosis, inflammatory diseases including atherosclerosis, as well as infectious diseases, obesity, diabetes, and various types of cancer. However, new data suggest that the scope of CCL2's functions may extend beyond its original characterization as a chemoattractant. Emerging evidence shows that it can impact leukocyte behavior, influencing adhesion, polarization, effector molecule secretion, autophagy, killing, and survival. The direction of these CCL2-induced responses is context dependent and, in some cases, synergistic with other inflammatory stimuli. The involvement of CCL2 signaling in multiple diseases renders it an interesting therapeutic target, although current targeting strategies have not met early expectations in the clinic. A better understanding of how CCL2 affects immune cells will be pivotal to the improvement of existing therapeutic approaches and the development of new drugs. Here, we provide an overview of the pleiotropic effects of CCL2 signaling on cells of the myeloid lineage, beyond chemotaxis, and highlight how these actions might help to shape immune cell behavior and tumor immunity.

## Introduction

Monocyte chemoattractant protein-1 [MCP-1, chemokine nomenclature: C–C motif chemokine ligand 2 (CCL2)] is a member of the chemokine family, a collection of small, secreted, chemotactic cytokines, named after their best known function of attracting cells (1). CCL2 is expressed by a variety of cells, such as endothelial cells, smooth muscle cells (2), fibroblasts (3), epithelial cells (4), mesangial cells (5), astrocytes (6), T cells (7) and tumor cells (8, 9), as well as by myeloid cells (see Table 1). CCL2 expression is inducible, triggered upon exposure to inflammatory stimuli, such as interleukin-1, interleukin-4, interleukin-6 (IL-1, IL-4, and IL-6), tumor necrosis factor α (TNFα), transforming growth factor β (TGFβ), lipopolysaccharide (LPS), interferon γ (IFNγ), platelet-derived growth factor (PDGF), vascular endothelial growth factor (VEGF), macrophage colony-stimulating factor (M-CSF), and granulocyte-macrophage colony-stimulating factor (GM-CSF) (29–33). It is found in the circulation, where it has been suggested as a diagnostic biomarker of breast cancer and prostate cancer (34, 35), as well as in tissues (www.proteinatlas.org) (36), where it attracts leukocytes to sites of infection or injury to mediate defense and repair.

**Table 1:** Expression of CCL2 by myeloid cells.

**Cell type**	**Stimulant**	**Readout**	**References**
h CD11b+cells	IL-6	Antibody array	(10)
THP-1 (monocyte cell line), THP-1 derived macrophages (M2 differentiated with PMA), primary h macrophages (adherent fraction of PBMCs)	LPS, TNFα	RT-PCR, ELISA	(11)
THP-1 derived macrophages (M2 differentiated with PMA)	Coculture with MCF10A tumor cells	Cytokine array, RT-PCR	(12)
m BMDMs (differentiated in L-cell CM)	LPS, IFNγ+LPS	RT-PCR	(13)
RAW264.7 (macrophage cell line)	LPS	RT-PCR	(14)
m synovial macrophages (adherent fraction of synovial cells)	Tenascin-C, osteopontin	RT-PCR	(15)
m PEMs (adherent fraction)	Coculture with MDA-MB 231 tumor cells	RT-PCR	(16)
m PEMs (M2 differentiated with M-CSF)	4T1 cancer-cell-derived GM-CSF	Northern blot	(17)
m microglial cells	Fragment of amyloid β-protein Aβ(25–35)	Northern blot	(18)
h female reproductive tract DCs (positive selection from endometrium tissue)	Human immunodeficiency virus	Multiplex assay	(19)
h DCs (from PBMCs, differentiated with IL-4 and GM-CSF)	Dengue virus	RT-PCR	(20)
h DCs (from PBMCs, differentiated with IL-4 and GM-CSF)	TLR3, TLR4, and TLR8 agonists	ELISA	(21)
h lung mast cells (isolated by elutriation)	rhSCF	Northern blot	(22)
HMC-1 (mast cell line)	rhSCF	ELISA	(22)
LAD 2 (mast cell line)	Complement factor C3a or C5a	ELISA	(23)
HMC-1 (mast cell line)	fMLP (bacterial-derived peptide)	RNase protection assay	(24)
h neutrophils	LPS, IFNγ, LPS+IFNγ, IFNγ+TLR2 ligands (LTA or Pam3CSK4), TNFα, TNFα+IFNγ	ELISA	(25)
h neutrophils	PMA, LPS, PAM3CSK4 (TLR2 ligand), FSL-1 (bacterial derived TLR2/6 agonist)	ELISA	(26)
RBL-2H3 (rat basophil cell line)	fMLP or C3a or C5a	ELISA	(27)
h eosinophils	CB+C5a, CB+fMLP, CB+PAF, GM-CSF, Ionomycin, PMA, IL-5+CB+C5a, IL-5+CB+PAF, CB+C5a, IL-5+C5a, IL-5+C5a+CB	ELISA	(28)

*This table provides an exemplary overview of the different types of myeloid cells and their ability to secrete CCL2. The stimulant and the readout are provided. BMDM, bone-marrow-derived macrophage; CB, cytochalasin B; CM, conditioned medium; DC, dendritic cell; fMLP, N-formylmethionine-leucyl-phenylalanine; h, human; m, murine; PAF, platelet-activating factor; PBMC, peripheral blood mononuclear cells; PEM, peritoneal exudate macrophage; PMA, 2-O-tetradecanoylphorbol-13-acetate; rhSCF, recombinant human stem cell factor; TLR, toll-like receptor*.

CCL2 was first discovered as a chemoattractant for monocytes (37, 38), but it also attracts T cells (39), B cells (40), natural killer cells (41), basophils (42), macrophages (43), dendritic cells (DCs) (44), myeloid-derived suppressor cells (MDSCs) (45), and neutrophils under very specific conditions (46). CCL2 signals to these target cell types by binding to and activation of the seven transmembrane G-protein-coupled receptor C–C chemokine receptor type 2 (CCR2) (47). Several intracellular downstream signaling cascades of CCR2 are known. Among these, activation of JAK2/STAT3 signaling (48), MAP kinase signaling (49, 50), and PI3K signaling (50, 51) are involved in promoting cell migration, as well as phospholipase-C-mediated calcium release (49, 52). In addition, CCL2 can bind to the atypical chemokine receptors ACKR1 and ACKR2 (53). These receptors belong to a different category of chemokine receptors, as they are not directly coupled to G proteins (54). The atypical chemokine receptors participate in the shaping of chemokine gradients and therefore act as regulatory components of chemokine networks (54, 55). To add to the complexity, CCL2 is also able to interact with the glycosaminoglycan chains of proteoglycans. It binds with varying affinities to different types of glycosaminoglycans, such as heparan sulfate, heparin, and dermatan sulfate (56, 57), which serve as coreceptors responsible for chemokine immobilization, presentation to leukocytes, and structural activation, i.e., oligomerization (58, 59).

During physiological host defense, for example upon tissue injury or infection, CCL2 expression is induced by inflammatory stimuli and promotes extravasation of effector cells from the blood stream across the endothelium (60, 61). However, aberrantly increased CCL2 expression is responsible for sustaining and exacerbating cell recruitment and the resultant inflammation. CCL2 has been implicated in the pathogenesis of conditions, such as rheumatoid arthritis (RA) (62), atherosclerosis (63), multiple sclerosis (64), diabetes (65), congestive heart failure (66), and many others [discussed comprehensively in (67, 68)]. In various types of cancer, such as breast (69), prostate (70), colorectal (71), or pancreatic (72) cancer, CCL2 recruits immune cells to the tumor microenvironment, additionally acting on stromal and tumor cells to modulate angiogenesis, metastasis, and tumor cell proliferation (45, 69, 73–75), comprehensively reviewed in Borsig et al. (76) and Lim et al. (77). Hence, a range of different targeting approaches has been developed with the goal of inhibiting CCL2's role in disease aggravation. To date however, therapeutic blockade of CCL2 has not been crowned with success. One potential reason for unexpected side effects and lack of efficacy observed in the clinic could be the emerging complexity of CCL2's effects on immune cell behavior. A detailed discussion of CCL2-targeting is provided in *Discussion and Conclusions* of this review.

In the past few years, more and more functions of chemokines have been discovered. An overview of all chemokines and their impact on leukocyte behavior can be found in Lopez-Cotarelo et al. (78). Several chemokines have been discussed in detail recently in a special issue of *Cytokine* (79). Furthermore, the specific impact of CCL2 on T cells (31, 80) and NK cells (81) has already been reviewed. Here, we focus on the molecular and cellular processes induced by CCL2 in myeloid cells beyond chemotaxis. Emerging evidence highlights a role for CCL2 not only in attracting cells but also affecting them functionally and morphologically. Understanding CCL2's potential impact on myeloid cells will contribute to deciphering disease pathogenesis and could therefore improve therapeutic targeting strategies.

**Figure 1:**
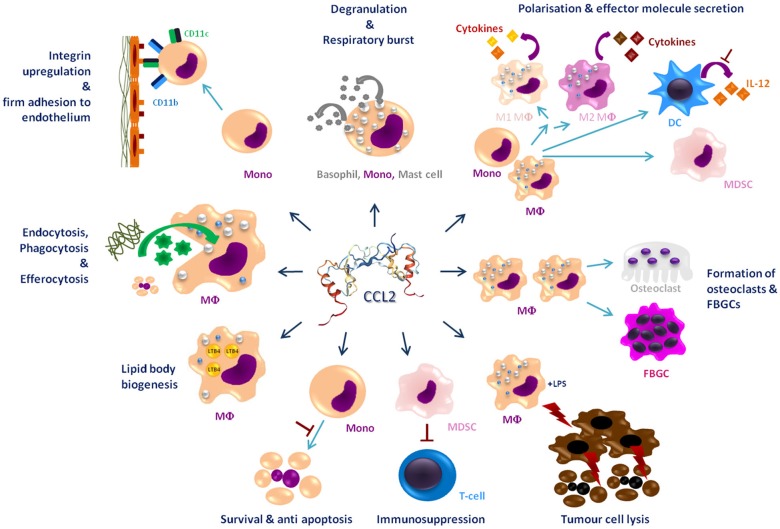
Graphical overview of the effects of CCL2 on myeloid cells apart from migration. CCL2 [structure PDB 1DON (131)] may impact a large number of myeloid cells including, monocytes (Mono), macrophages (MΦ), dendritic cells (DC), mast cells, osteoclasts, foreign body giant cells (FBGCs), myeloid-derived suppressor cell (MDSC), and basophils. It causes a wide range of different effects on these cells labeled in dark blue headings. Red⊥ -symbols describe inhibition. Bent arrows describe secretion or intake of molecules; discriminated by the direction of the spearhead.

This review summarizes the effects of CCL2 on myeloid cells and is divided into subsections detailing its different functions. In addition, Tables 2–**5** provide a more detailed overview of the experiments regarding the source of CCL2, the modes of blocking CCL2/CCR2, the models, and the readouts. They are grouped according to myeloid cell types to provide an additional perspective on CCL2's functions. Furthermore, Figure 1 shows a schematic graphical depiction of the multiple effects of CCL2 on myeloid cells.

**Table 2:** CCL2's effects on monocytes.

**Effect on monocytes**	**Source of CCL2 or CCL2/CCR2 blocking/KO**	**Cells/model**	**Methods and results**	**References**
**MATURATION/DIFFERENTIATION/CYTOKINE PRODUCTION**				
Influences cytokine production	CCL2 purified from U-105 MG CM	h monocytes in serum-free conditions plated on 2% agarose	Proinflammatory cytokines IL-6↑, IL-1↑, TNF (=) (MH60 cell proliferation assay, D10 cell proliferation assay, L929 cytopathic assay)	(82)
	rCCL2	h CD11b+ cells treated with rCCL2 in serum-free medium	Proinflammatory cytokine IL-6↑ (cytokine and inflammation arrays)	(10)
	rCCL2, pertussis toxin	h monocytes preincubated with medium +/– rCCL2, then stimulated with SAC and IFNγ	Preincubation with rCCL2: cytokine IL12p70↓ (ELISA) IL-12p35↓, IL-12 p40↓ (RT-PCR), with pertussis toxin pretreatment: IL12p70 (=) (ELISA)	(83)
	rCCL2	TPA-preactivated THP-1 cells stimulated in serum-free conditions with +/– rCCL2.	Proinflammatory cytokine TNFα↑ (ELISA)	(84)
	Intrinsic CCL2 of monocytes, anti-CCL2 Ab	h monocytes (GG or AA genotype in −2518) + *M. tuberculosis* H37Rv sonicate +/– anti-CCL2 Ab	GG vs. AA genotype: CCL2↑, IL-12p40↓, GG genotype + anti-CCL2 Ab: IL12-p40↑ (ELISA)	(85)
Enhances maturation into M2 macrophages	rCCL2	h CD11b+ after isolation and rCCL2 stimulation in serum-free conditions	M2 macrophage marker in CD14+ cells: CD206↑(FC)	(10)
**INTEGRIN EXPRESSION AND ACTIVATION, ARREST**				
Induces integrin expression	CCL2 purified from U-105 MG CM	h monocytes stimulated with CCL2	Integrin expression: CD11a (=), CD11b↑, CD11c↑, CD18↑ (FC), Selectin LAM-1 (=) (FC)	(82)
	rCCL2	h monocytes stimulated with rCCL2	Integrin expression: CD11a (=), CD11b↑, CD11c↑, CD18↑, VLA-4α (=) (FC), general monocyte markers unaffected: CD14 (=), CD15 (=) (FC), adhesion↑ (adhesion assay)	(86)
Increases firm adhesion and arrest	wt and CCL2 KO mice upon inflammation, rCCL2	Labeled WEHI78/24 cells injected through femoral artery catheter and PLNs HEV analyzed	Inflamed PLN HEVs: arresting cells↑, CCL2 KO mice: arresting cells↓ CCL2 KO mice + rCCL2: arresting cells ↑ (intravital microscopy)	(87)
	rCCL2	Flow chamber assay with HUVEC monolayer (transduced with E-selectin adenovirus) and h monocytes	Adhesion↑ (videomicroscopy, quantification per HPF)	(88)
	Inflamed endothelial cells, anti-CCL2 Ab, CCL2 antisense oligomer, CCL2 antagonist, anti-CCR2 Ab, integrin-blocking Abs	Flow chamber assay with TNF- activated HPAEC monolayer and h monocytes	Upon blocking CCL2 or CCR2: adhesion↓, upon blocking integrins: adhesion↓ (videomicroscopy, quantification per HPF)	(89)
Induction of arachidonic acid release	rCCL2, anti-CCL2 antiserum, pertussis toxin, phospholipase A_2_ inhibitors (p-bromophenacyl bromide, manoalide)	Prelabeled h monocytes and THP-1 cells stimulated with rCCL2 +/– pre-treatment with pertussis toxin or antiserum, migration assay toward rCCL2 in presence of phospholipase A_2_ inhibitors	[3H]Arachidonic acid release: with rCCL2↑, with anti-CCL2↓, with pertussis toxin↓ (liquid scintillation spectrometry), Migration toward rCCL2: in presence of phospholipase A2 inhibitors ↓ (modified Boyden Chamber migration assay)	(90)
**ENHANCEMENT OF SURVIVAL**				
Enhances survival	rCCL2	h CD11b+ cells treated with rCCL2 under serum deprivation	Antiapoptotic proteins↑ (cFLIP_L_↑, Bcl-2↑, Bcl-X_L_↑), caspase cleavage↓ (caspase 8, −3, −6, −7 cleavage↓), Lamin A cleavage↓ (WB), survival↑ (WST-1 cell viability assay), apoptotic cells ↓ (FC)	(10)
**ENHANCEMENT OF HOST DEFENSE, CELLULAR CLEANUP**				
Hyperactivates autophagy	rCCL2	h CD11b+ cells treated with rCCL2 under serum deprivation	Microtubule-associated protein cleavage: LC3 cleavage↑ (WB)	(10)
Induces respiratory burst	rCCL2	h monocytes exposed to rCCL2	NADPH oxidase activity↑ (H_2_O_2_ formation)	(91)
	Purified CCL2 from TNF-stimulated fibrosarcoma cell line 8387	h monocytes exposed to purified CCL2	*N*-acetyl beta-d-glucosamininase release↑ (release assay), superoxide anion release ↑ (release assay)	(92)
Tumor cell killing/growth inhibition	Purified CCL2 from supernatant of THP-1 cells stimulated with LPS, silica, and hydroxyurea	h monocytes exposed to purified CCL2 and added tumor cell suspension	Growth of tumor cell lines HT29, A375, HTB, MCF7, HTB 88 ↓ ([3H] thymidine incorporation assay)	(37)
	CCL2-expressing CHO cells (CCL2 transfected) *in vivo*	m tumor model by injection of CCL2 expressing/non-expressing CHO cells or coinjection of CCL2 expressing and non-expressing CHO cells and HeLa cells	CCL2 expressing cells: tumor formation↓ coinjection of CCL2 expressing and non-expressing cells: tumor formation ↓(histology)	(93)
	Glioblastoma lines HBT20 and HBT28 (CCL2 transfected)	CCL2-expressing HBT20 and HBT28 cell lines cocultured with h monocytes activated with LPS	Tumor cell lines + activated monocytes: cytostasis↑ ([3H]thymidine deoxyribose uptake cytostasis assay)	(94)

*Ab, antibody; CHO, Chinese hamster ovary cell; CM, conditioned medium; FC, flow cytometry; h, human; KO, knockout; HEV, high endothelial venule; HPF, high-power field,; PLN, peripheral lymph node; RT-PCR, reverse transcription PCR; rCCL2, recombinant CCL2; SAC, Staphylococcus aureus Cowan strain 1; SN, supernatant; TPA = PMA, tetradecanoylphorbol-acetate; WB, Western blot; ↑, upregulation; ↓, downregulation; (=), level stays the same*.

## Effects of CCL2 on Myeloid Cells

### CCL2: Not Simply a Guidance Cue During Monocyte Extravasation

Chemotaxis, classically defined as directional cell movement along a gradient of increasing signal substance concentration (132), is the best studied function of CCL2. Monocytes circulate in the blood stream and extravasate into the tissue where they give rise to macrophages and DCs, and they actively participate in innate immune defense (133). Monocyte extravasation comprises various stages including capture, slow rolling on the endothelium, firm adhesion/arrest, intraluminal crawling, and transmigration (134). During this process, CCL2 is presented by the endothelium via proteoglycans as a guidance cue for extravasation, which subsequently activates G-protein-coupled receptor-mediated transmigration events. In the tissue, it further guides monocytes along a chemokine gradient to the location of insult (135, 136). A pivotal role for CCL2 in attracting blood monocytes has been well established in *in vitro* (38) and *in vivo* studies. For instance, injecting recombinant rat CCL2 into rat skin intradermally induced intra- and extravascular accumulation of monocytes 3 h after injection (137). In an animal model of type II diabetes, the treatment of a diabetic wound with CCL2 increased monocyte/macrophage infiltrate into the wound tissue (138). Monocyte infiltration toward CCL2-producing sites was also detected in transgenic models, where the expression of CCL2 was targeted to distinct organs via specific promotors, namely, the thymus (via Lck) or central nervous system (via myelin basic protein) (139), pancreatic islets (via insulin) (140), or type II alveolar epithelial cells (via surfactant protein C) (141). Moreover, blocking antibodies against CCL2 have been investigated in a variety of disease models *in vivo*, e.g., in hepatocellular cancer, where intraperitoneal (i.p.) injection of an anti-CCL2 antibody reduced inflammatory myeloid cells in the liver compared to control, or in a zymosan-induced peritonitis model, where coadministration of an anti-CCL2 antibody with zymosan i.p. significantly reduced monocyte accumulation in the peritoneal cavity (60). Likewise, studies investigating the role of CCL2 via CCL2 knockout mice during inflammatory responses via i.p. injection of LPS, zymosan, or thioglycollate also showed a significant reduction in monocyte infiltration to the peritoneal cavity (142, 143). The significant reduction in monocyte infiltration in all these different *in vivo* models indicates a non-redundant role of CCL2 as monocyte chemoattractant.

However, at the leukocyte/endothelial interface, CCL2 is not merely acting as guidance cue; it also increases firm adhesion of human monocytes to vascular endothelium under flow conditions, monitored via video microscopy (88). This effect was activated in response to endothelially produced CCL2 (89) and mediated by specific monocytic cell surface receptors. CD11/CD18 heterodimers form the β2 integrin family, each consisting of a different α-subunit (α_L_–CD11a, α_M_–CD11b, α_X_–CD11c, and α_D_–CD11d) paired with a common β-subunit (CD18 or β2) (144). These integrins mediate cell adhesion and chemotaxis, causing rearrangement of the cytoskeleton (145). Intracellular signals, among them chemokine receptor activation, cause structural changes in integrins that lead to enhanced affinity and avidity toward their extracellular ligands (“inside–out signaling”). Subsequent extracellular ligand binding to integrins then induces downstream signaling cascades (“outside in signaling”), which impacts immune cell recruitment, immune cell interactions, and immune cell signaling (144). It was discovered that CCL2 increased surface protein expression of the α-chains of two members of the β2 family of integrins, CD11b and CD11c, and their common β-chain CD18, whereas no effect on the expression of CD11a, VLA-4α, or the selectin LAM-1 was observed in human monocytes (82, 86). Enhanced expression of CD11b and CD11c correlated with enhanced binding of monocytes to endothelial cells (86). Moreover, it was shown that CCL2 increased monocyte adhesion to TNFα-stimulated human pulmonary artery endothelial cells under flow conditions, in a β2-integrin-dependent manner (89). In addition, Yi et al. found that activated integrins are polarized at the leading edge of monocytes within 2 min of CCL2 addition (146). Likewise, CCL2 produced *in vivo* upon inflammation in peripheral lymph nodes, or CCL2 injected into CCL2 knockout mice, enhanced integrin-mediated arrest of labeled WEHI78/24 (murine monocytoid) cells injected through the femoral artery and visualized via intravital microscopy, whereas their propensity to roll was unaffected by the presence of CCL2 (87).

CCL2 can also induce arachidonic acid release in human monocytes, which has been shown to be involved in adhesion and induction of the chemotactic response, in a pertussis-toxin-sensitive manner (90, 147). The involvement of arachidonic acid in monocyte adhesion was shown using manoalide and bromophenacyl bromide, phospholipase A_2_ inhibitors, which blocked release of arachidonic acid and impaired cell adhesion in human monocytes *in vitro*. This effect was partially reversed by the addition of arachidonic acid (148). Furthermore, the same inhibitors led to a decreased migration of human monocytes *in vitro* in a microchemotaxis chamber migration assay toward CCL2 (90). In addition, CCL2 can activate monocytes, and depending on the experimental setup, different outcomes were shown for monocyte stimulation with CCL2. Human monocytes upregulated IL-1 and IL-6 upon CCL2 stimulation, whereas TNFα was unaffected (82). The increased expression of IL-6 upon CCL2 treatment of human monocytes was also confirmed by Roca et al. (10). Neumark et al. found that CCL2 was able to promote the secretion of TNFα from monocytic THP-1 cells but only after preactivation with tetradecanoylphorbol acetate (TPA = PMA) (84).

The data in this section show that CCL2 not only serves as a chemotactic guidepost during monocyte extravasation but that it can also control cell adhesion by modulating integrin expression and localization, as well as by arachidonic acid release. Moreover, CCL2 may also play a role in activating monocytes to produce inflammatory cytokines. It is of note that CCL2 responses are not isolated phenomena but can be viewed as successive or even simultaneous events because responsiveness to migratory cues is often tightly linked to signals that regulate cell activation or differentiation (31).

### CCL2 Induces Context-Specific Macrophage Polarization and Cytokine Secretion

Macrophages are phagocytic cells, which are also a major source of cytokines during inflammation. Highly heterogeneous, their extraordinary functional plasticity enables them to respond to diverse microenvironmental stimuli (149). The traditional division into M1 (classically activated, pro-inflammatory) and M2 (alternatively activated, anti-inflammatory) provides a simple classification of macrophage behavior, although in reality, a multitude of different activation states exists (150). However, this classification is useful in providing an overview of the diverging cellular responses to CCL2. Multiple cytokines have been described in the complex network of macrophage polarization, and their roles have been successively revealed (151, 152). The M1 phenotype is characterized by increased expression of cytokines, such as TNFα, IL-1β, IL-6, and IL-12; increased expression of surface receptors, such as Fcy-RI, II, and IIIA; as well as the production of reactive oxygen species (ROS) and reactive nitrogen species (151, 153). On the other hand, M2 polarization shows higher expression of IL-10, MRC1/CD206, Ym1, and Arg1 (151, 154), alongside an increasing list of differentially expressed molecules [reviewed in (150, 155)]. A role for a number of chemokines in influencing macrophage polarization in inflammatory conditions has been described (152), including CCL2. Our current understanding of CCL2's impact on macrophage polarization suggests both an impact on M1 and M2 polarization, which appears to be context dependent (see Tables 3, **5**).

**Table 3:** Summary of CCL2's effects on macrophages.

**Effect on macrophages**	**Source of CCL2 or CCL2/CCR2 blocking/KO**	**Cells/model**	**Methods and results**	**Disease**	**References**
**MATURATION/DIFFERENTIATION/CYTOKINE PRODUCTION**					
CCL2/CCR2 deficiency enhances M2 polarization	Cell-derived (after transfection of ICAM-1 siRNA, miR124 mimic)	RAW264.7 macrophages (transfected), porcine alveolar macrophages (transfected)	ICAM-1 siRNA: M1 markers iNOS↑, CD86↑, IL-6 ↑, M2 markers Ym1 (= /↓), Mrc-1 (=), IL-10 (=) miR124 mimic: M1 markers iNOS↓, CD86 (= /↓), IL-6↓, M2 markers Ym1↑, Mrc-1↑, IL-10 (= /↑) (RT-PCR)	Acute lung injury	(95)
	CCR2 KO HFD mice	Adipose tissue macrophages from high-fat diet (HFD) mice (+/–CCR2 KO)	HFD CCR2 KO vs. HFD wt: M2-markers IL-10↑, Ym1↑, Arg1↑, Mrc2↓, Mgl1↑, Mgl2↑ (RT-PCR)	Obesity	(96)
	CCL2 KO mice	Lipoatrophic diabetic A-ZIP-Tg mice (+/– CCL2 KO), livers analyzed	CCL2 KO vs. wt: M2 markers Arg1↑, Ym1↑, TGFβ↑ (RT PCR), phosphorylation ERK-1/2, and p38MAPK↓ (WB)	Insulin resistance and hepatic steatosis	(97)
Enhances M1 polarization	rCCL2	Starved RAW264.7 macrophages treated with CCL2	M1 marker TNFα↑, M2 marker Arg1↓ (RT-PCR)	Abdominal aortic aneurysm	(98)
	No info about CCL2, Rho inhibitor Fasudil	Alveolar macrophages and BMDMs + CCL2 +/– LPS +/– Fasudil	M1 markers: iNOS↑, IL-6↑, IL-1β↑, effect diminished by Fasudil (RT-PCR)	Allergic inflammation	(99)
Enhances M2 polarization	Anti-CCL2 Ab	h monocytes polarized with M-CSF +/– anti-CCL2 Ab	anti-CCL2 Ab vs. IgG: GM-MΦ-markers IL23A↑, IL12B↑, TNF↑, CCL1↑, SERPINE1↑, EGLN3↑, INHBA↑, ALDH1A2↑, M-MΦ-markers IGF1↓, STAB1↓, CMKLR1↓, SLC40A1↓, FOLR2↓, HMOX1↓, CD163↓, HTR2B↓ (RT-PCR)	/	(100)
	SN from chitin-exposed epithelial cells, anti-CCL2 Ab, rCCL2, CCR2 KO mice, CCL2 KO mice	AMJ2-C11 m macrophages + SNs from chitin-exposed LA-4 m lung epithelial cells +/– anti-CCL2 Ab or + rCCL2, BALF CD11c+ of KO mice vs. wt after chitin-treatment	SN+anti-CCL2-Ab: M2 marker Arg1↓, rCCL2: Arg1 (=), CD11c+ of CCR2KO: M2 markers Arg1↓, CCL17↓, CCL22↓(RT-PCR), M2 markers CCL17↓, CCL22↓, Ym1↓ (ELISA) CCL2KO: CCL7↑	Allergic lung inflammation	(101)
Osteoclast formation, FBGC formation	CCL2 and CCR2 KO, rCCL2	Bone marrow derived CCL2 and CCR2 KO osteoclasts (differentiated in M-CSF and RANKL) and FBGCs (differentiated in GM-CSF and IL-4) *in vitro*, osteoclasts *in vivo*	KO: cell count, nuclei count and cell size of TRAP stained cultures of osteoclasts and FBGCs ↓, rescued by rCCL2 KO: cell number, and number of nuclei in TRAP stained bone marrow sections ↓	/	(102)
	Dominant negative CCL2 mutant protein 7ND or CM of 7ND transfected HEK293T cells	Cord blood derived osteoclasts (differentiated from CD14+ cells by GM-CSF, IL-3, SCF to form CFU-GM cells; then treated with RANKL and M-CSF to form osteoclasts) treated with 7ND	NFATc2↓, CALM1↓, JUN↓ (RT-PCR), osteoclast formation↓ (TRAP staining and cell counting)	/	(103)
	CCL2 KO, anti-CCL2 Ab, rCCL2	Bone-marrow-derived CCL2 KO and wt osteoclasts (differentiated in M-CSF and RANKL), primary osteoclasts from CCL2 KO and wt mice	CCL2 KO osteoclasts: osteoclast formation↓ (cell count of TRAP pos), c-Fms↓, RANK↓(RT-PCR) Ab treated wt osteoclasts: c-Fms ↓(FC) CCL2 treated wt and CCL2 KO osteoclasts: c-Fms↑, RANK↑ (RT-PCR), CCL2 KO osteoclasts: pERK↓ (WB), CCL2 KO primary mBM: F-actin formation ↓ (rhodamine-phalloidin stain)	/	(104)
	rCCL2, anti-CCL2 Ab, CCL2 KO	BMDMs from wt and CCR2 KO mice (differentiated in M-CSF and RANKL)	rCCL2-treated wt BMDMs: osteoclast number↑ (cell counts of TRAP pos), mAb-treated wt BMDMs: osteoclast number↓ (cell counts of TRAP pos), rCCL2 treated wt BMDMs: RANK↑ (FC), CCL2 KO BMDMs: RANK↓ (FC), CCL2 KO mice: bone mass ↑ (bone mass measurement), NF-κB and ERK1/2 signaling ↑ (RT-PCR)	/	(105)
**PRIMING OF CELLS TO RESPOND TO SUBSEQUENT INFECTION**					
Enhances response to subsequent infection/inflammation	rCCL2	rCCL2 pre-treated BMDMs (matured in L-cell CM) +/– IFNγ +/– LPS	rCCL2+IFNγ+LPS: M1 markers iNOS↑, TNFα↑, rCCL2: ERK1/2 phosphatase Dusp6↓, Dusp6-targeting miRNA miR9↑ (RT-PCR) rCCL2+IFNγ+LPS: M1 marker TNFα↑ (Luminex) rCCL2+IFNγ+LPS: pERK/tERK ratio ↑ (WB)	Inflammation	(13)
	CCL2 KO mice, rCCL2	MLM prepared from wt mice and CCL2 KO mice (+/– burn injury), +/– stimulated with heat-killed *E. faecalis*, +/– rCCL2; culture fluids analyzed	MLM from burned CCL2 KO mice vs. wt: CCL1 (=), CCL17↓, CCL13↓, MLM from wt mice with rCCL2: CCL1 (=), CCL17↑, CCL13↑, MLM from burned CCL2KO mice treated with rCCL2 vs. untreated: CCL1 (=), CCL17↑, CCL13↑ (ELISA)	Severe burn injury	(106)
	SIRS mouse sera (naturally containing CCL2), rCCL2, anti-CCL2 Ab	PEMs cultured with SIRS or normal mouse sera +/– anti-CCL2 Ab or rCCL2, PEMS isolated after +/- SIRS sera or anti-CCL2 Ab injection	SIRS vs. normal sera: M2 marker CCL17↑ (ELISA), M2 marker mannose receptor↑ (RT-PCR) PEMs of mice + SIRS sera: CCL17↑, with anti-CCL2-Ab: CCL17↓, PEMs of normal mice + rCCL2: CCL17↑ (ELISA)	Systemic inflammatory response syndrome (SIRS)	(107)
	rCCL2	h differentiated GM-Φ cultured with LPS +/– rCCL2	LPS+rCCL2: IL-10↑, IL-6(=), IL12p40(=), TNFα(=) (ELISA)	/	(100)
	CCR2 KO mice	GM-F from wt and CCR2 KO mice cultured with LPS	CCR2 KO: IL-10 (=), IL-6↑, TNFα↑, CCL2↑ (ELISA)	/	(100)
	CCL2 KO, CCR2 KO mice	LPS stimulation of M-CSF differentiated BMDMs	M1 markers: CCL2 KO: TNFα↑, CCR2 KO: TNFα(=), CCL2 KO and CCR2 KO: iNOS↑, CCL2 KO and CCR2 KO: IL-12↑, M2 marker: CCL2 KO and CCR2 KO: IL-10↓	Retinal para-inflammation	(108)
	Anti-CCL2 Ab, rCCL2	Type 2 (SEA) pulmonary granuloma Φ of mice-treated +/– anti-CCL2 Ab, CFA or oil-elicited PEMs stimulated +/– rCCL2 + LPS	rCCL2: IL12↓, anti-CCL2-Ab: IL-12↑(ELISA)	Lung granuloma	(109)
**ACTIVATES CYTOTOXICITY, PHAGOCYTOSIS, EFFEROCYTOSIS**					
Increases macrophage cytotoxicity	rCCL2	rCCL2-primed RAW264.7 macrophages and primary mouse aortic SMCs in co-culture	apoptosis of SMCs↑ (Annexin V-PE/7-AAD apoptosis kit), CCL2-priming of RAWs: FasL↑ (RT-PCR), cleaved caspases↑, FasL↑(WB)	Abdominal aortic aneurysm	(98)
Increases efferocytosis of apoptotic cells	rCCL2, *in vivo*	m AMs, PEMs, and J774 macrophage cell line *in vitro* treated with rCCL2, m AMs +/–rCCL2 *in vivo*	Phagocytic index upon rCCL2 treatment ↑ (*in vitro* and *in vivo* phagocytosis assays)	Acute lung inflammation	(110)
Increases phagocytosis and endocytosis	CCL2 KO, CCR2 KO mice	BMDM (M-CSF stimulated) from KO vs. wt mice	Phagocytosis: CCR2 KO↓, CCL2 KO↓ (*E. coli* phagocytosis assay), Endocytosis: CCL2KO↓, CCR2KO↓ (dextran endocytosis assay)	Retinal para-inflammation	(108)
Increases extracellular matrix turnover	CCR2 KO	Macrophages of CCR2 KO mice	CCR2 KO: number of collagen endocytosing Ly6C+ and Ly6C- macrophages↓(FC), number of Dex^low^ macrophages ↓ (FC), number of total and Ly6C+ fibrin endocytosing macrophages↓ (FC)	/	(111)
**ENHANCES SURVIVAL, PROLIFERATION**					
Anti-apoptotic, increases survival and proliferation	NFAT5 shRNA, rCCL2, anti-CCL2 Ab	h RA-SF macrophages, m splenic macrophages, PEMs	Apoptosis: RA-SFΦ: with NFAT5 shRNA↑, with NFAT5 shRNA + rCCL2 (=), splenic Φ + anti-CCL2 Ab↑ (FC), PEMs + anti-CCL2 Ab: proliferation↓ (MTT assay)	Rheumatoid arthritis	(32)
	CCL2 KO mice, rCCL2	EdU administration via drinking water in wt and CCL2 KO mice receiving HFD, treatment of visceral AT explants of ob/ob mice with rCCL2 and EdU *ex vivo*	Proliferation rate: AT Φ of CCL2 KO↓, rCCL2 treatment of explants↑ (FC: percentage of EdU+ macrophages)	Obesity associated adipose tissue inflammation	(112)
**CYTOKINE PRODUCTION**					
Cytokine production	Anti-CCL2 Ab	h M-GSF differentiated Φ generated in presence of +/– anti-CCL2 Ab	CCL2-blocking: IL6↑ (ELISA and RT-PCR)	/	(100)
	CCR2 KO mice	GM-Φ from CCR2KO mice compared to wt	CCR2KO: M2 markers Htr2b↓, IL10↓, M1 markers TNF (=), Inhba (=), CCR7(=)(RT-PCR)	/	(100)
	CCR2 KO mice	M-Φ from CCR2KO mice compared to wt	CCR2KO: M2 markers Folr2↓, Htr2b↓, IL10↓, M1 markers TNF↑, Inhba↑, CCR7 (=)	/	(100)
	rCCL2, MEK inhibitor PD98059	PEMs stimulated *in vitro* with rCCL2 +/– PD98059	Pro-inflammatory M1 marker: TNFα↑, addition of PD98059: TNFα↓ (RT-PCR and L929 cell assay for TNF activity)	Inflammation and tumor growth	(113)
	rCCL2, Anti-CCL2 Ab	Neuron + m microglia co-culture +/– rCCL2; Induction of thiamine deficiency in cocultures +/– anti-CCL2 Ab	rCCL2: microglia activation↑, anti-CCL2 Ab: microglia activation (=)(microscopy), rCCL2: TNFα↑, IL1β ↑ (RT-PCR)	Neurodegeneration	(114)
**ACTIVATION OF LIPID BODY BIOGENESIS**					
Activation of lipid body biogenic and functional machineries	CCL2 and CCR2 KO mice, anti-CCL2 Ab, rCCL2	PEMs from wt and KO mice +/– LPS or +/– CLP, +/– anti-CCL2 Ab or +/– rCCL2, analysis of cell-free SN from peritoneal lavage	Lipid bodies per cell: CCL2 KO: ↓, CCR2 KO:↓ wt mice + anti-CCL2 Ab:↓, CCL2 KO+ rCCL2:↑ (microscopy), newly synthesized LTB4: CCL2KO: ↓, CCR2KO:↓, wt mice + anti-CCL2 Ab:↓, CCL2 KO+ rCCL2:↑ (ELISA)	Infection-driven inflammatory disorders	(115)
	CCR2 KO mice, anti-CCL2 Ab	PEMs +/– anti-CCL2 Ab + oxLDL, *in vivo* injection of oxLDL in wt and KO mice + analysis of peritoneal wash	Lipid bodies/cell: anti-CCL2 Ab↓, CCR2 KO↓, Lipid body-associated protein: anti-CCL2 Ab: ADRP↓ (WB)	Atherosclerosis	(116)

*Ab, antibody; ADRP, adipose differentiation-related protein; AM, alveolar macrophage; BMDM, bone-marrow-derived macrophage; CFA, complete Freund's adjuvant; CM, conditioned medium; FBGC, foreign body giant cell; FC, flow cytometry; h, human; HFD, high-fat diet; KO, knockout; m, murine; MLM, mesenteric lymph node macrophage; oxLDL, oxidized low-density lipoprotein; PEM, peritoneal exudate macrophage; RT-PCR, reverse transcription PCR; rCCL2, recombinant CCL2; RA-SF, rheumatoid arthritis synovial fluid; SEA, soluble (Schistosoma mansoni) egg antigens; SIRS, systemic inflammatory response syndrome; SN, supernatant; SMC, smooth muscle cells; WB, Western blot; VAT, visceral adipose tissue; wt, wild type. ↑, upregulation; ↓, downregulation; (=), level stays the same; Φ, macrophage*.

Basal cytokine synthesis was comparable in unstimulated bone-marrow-derived macrophages (BMDMs) from wild-type, CCL2 knockout, or CCR2 knockout mice, in which low levels of inducible nitric oxide synthase (iNOS), IL-12, TNFα, and IL-10 gene expression were observed (108). Direct stimulation of cells with CCL2 favored M1 macrophage polarization in some reports, for example causing upregulation of TNFα and downregulation of Arg1 in the macrophage cell line RAW264.7 (98), increasing TNFα expression in peritoneal macrophages (113), or upregulating IL-1β, iNOS, and IL-6 messenger RNA (mRNA) levels in murine alveolar macrophages and BMDMs (99). Similarly, downregulation of CCL2 expression via overexpression of miR124 in RAW264.7 cells and porcine alveolar macrophages led to decreased M1 and enhanced M2 polarization without any additional stimulation, as measured by expression of M1 (iNOS, IL-6) and M2 (IL-10, MRC1, Ym1) markers. In RAW264.7 cells transfected with miR124 in the presence of LPS, the M1 phenotype could be rescued (95). Apart from Sodhi and Biswas, who confirmed that their CCL2 was endotoxin-free and showed that the effect of CCL2 stimulation could be blocked with an anti-CCR2 antibody (113), other studies cited here leave open the possibility that effects observed may be due to contaminating inflammatory stimuli such as LPS or harder to detect lipoproteins in recombinant protein preparations, or to other cellular changes caused by miR124 overexpression, and require confirmation that the effect was specific to CCL2. *In vivo* CCL2 knockout mice displayed an M2 phenotype, evidenced by elevated expression of Arg1, Ym1, and TGFβ, in the livers of lipoatrophic diabetic A-ZIP-Tg mice, and exhibited improved insulin resistance and protection from hepatic steatosis (97). Similarly, in adipose tissue macrophages of CCR2 knockout mice on a high-fat diet, M2 markers IL-10, Ym1, and Arg1 were upregulated compared to wild-type mice on a high-fat diet, with CCR2-null mice exhibiting similarly high levels of M2-like cells as found in lean wild-type mice (96). These studies hint at a contribution of CCL2 in driving M1 macrophage polarization under certain pathological situations, but a direct mode of action has yet to be established.

In contrast, an M2 polarizing function for CCL2 has been reported in several other studies. Stimulation of human CD11b+ myeloid cells with recombinant CCL2 led to an increase in CD14+/CD206+ cells, suggesting a polarization toward M2-type macrophages (10). Sierra Filardi et al. dedicated an elaborate paper to the elucidation of the CCL2/CCR2 axis involvement in macrophage differentiation/polarization by GM-CSF and M-CSF. They found that adding recombinant CCL2 during polarization of murine and human monocytes with M-CSF or GM-CSF increased M2 markers in several different settings *in vitro*. The use of an anti-CCL2 antibody in some of their *in vitro* polarization assays indicates a specific role for CCL2 in this polarization process. They also showed that this effect was recapitulated in CCR2 knockout mice. In these mice, the absence of CCR2 altered the M1/M2 ratio in the mouse peritoneum in a way that M2 peritoneal macrophages were almost absent compared to wild-type mice (100). These publications show a direct evidence of CCL2 on polarization *in vitro* and a dependence of the effect on CCR2. To back this up, *in vivo* studies using blocking antibodies or CCL2 transgenic mice will be required.

In a different experimental setup, chitin-induced CCL2 secretion by epithelial cells has been shown to result in CCR2-mediated M2 macrophage activation *in vitro*. The supernatant of epithelial cells lost the ability to induce the M2 marker Arg1 expression in macrophages in the presence of a CCL2 neutralizing antibody. However, recombinant CCL2 alone was not able to induce M2 polarization. Therefore, it appears that CCL2 is necessary but not sufficient to induce Arg1 expression in this experimental model (101). Knockout of CCR2 significantly decreased M2 markers expressed by CD11c+ cells isolated from bronchoalveolar lavage fluid (101). Moreover, upon knockout of CCL2, a compensatory increase in the expression of CCL7, which also signals via CCR2, was observed in whole lung homogenates that may obscure the role of CCL2 in macrophage M2 polarization. Therefore, CCL2 may act redundantly while CCR2 is required for chitin-induced M2 polarization in the lung. Although the CCL2-specific impact was shown via a blocking antibody *in vitro, in vivo* models including a CCL2-blocking antibody or transgenic mouse models are required to provide more clarity.

To elucidate the signaling mechanisms underlying macrophage polarization observed by addition of recombinant CCL2, different groups used blocking reagents or inhibitors. The CCL2 induced increase in TNFα expression in peritoneal exudate macrophages (PEMs) was shown to be mediated via activation of p42/44 MAPK and c-Jun and could be significantly reduced with the MEK inhibitor PD98059 (113). Stimulation of human macrophages with CCL2 increased the activation of p38, ERK1/2, MSK1/2, HSP27, JNK, and STAT5a/b (100). Moreover, in the murine A-ZIP-Tg CCL2 knockout model, CCL2 deficiency led to reduced ERK1/2 and p38 MAPK phosphorylation (97). In a different study, Gu et al. found that silencing the adhesion molecule ICAM-1, which positively modulates expression of the CCL2 mRNA binding miR124, increases CCL2 expression (95). As such, there is still a lot to uncover regarding the underlying signaling mechanisms. As many different intermediate activation states can occur in macrophages and a lot of fine-tuning is involved in the process of activation, it will be important to work toward a more detailed understanding about macrophage polarization and the molecules and signaling pathways involved in it.

In summary, these publications have shown that CCL2 may influence macrophage polarization and cytokine production. However, the potential of CCL2 to exert activating and polarizing effects on monocytes/macrophages *in vivo*, and the pathophysiological relevance of these actions, is still under debate. As described in *CCL2: Not Simply a Guidance Cue During Monocyte Extravasation*, a non-redundant role for CCL2 in mediating monocyte chemotaxis *in vivo* was demonstrated by blocking CCL2 via antibodies, CCL2 KO mice, injecting exogenous CCL2, and transgenic models in which CCL2 was overexpressed at specific organs. In these transgenic models (140, 141), monocyte infiltration was detected within the CCL2 producing organs, but monocytic activation (investigated via morphologic changes, retaining the expression of L-selection, which is normally shed upon inflammatory activation, or absence of respiratory burst) was unaffected. However, if the activating effect of CCL2 on monocytes/macrophages is transient, it is possible that it might have been missed in a transgenic model where chronic CCL2 stimulation occurs (141). It is also of note that in these publications, the authors did not take an in-depth look at cytokine synthesis and macrophage differentiation or polarization via macrophage marker expression on RNA or protein levels. The data summarized in this section (also see Tables 2, 3, 5) suggest a role for CCL2 in impacting activation and differentiation/polarization of macrophages. There are plenty of *in vitro* data including CCL2-blocking antibodies or cells from CCL2 knockout mice supporting this role. However, more *in vivo* data using CCL2 injections, CCL2-blocking antibody injections, and transgenic mouse models specifically looking at these effects would be helpful to confirm a direct effect of this chemokine and to rule out chemokine redundancy. Moreover, the effect of CCL2 is dependent on the activation and polarization state of the cells at the time of stimulation, and its role in favoring a particular polarization state is highly context dependent.

### CCL2 Primes Cells to Respond to Subsequent Infection

In addition to studies examining the effect of CCL2 on macrophage polarization, data are emerging which reveal that CCL2 may also have the capability of priming myeloid cells. The process of priming describes the exposure of a cell to a stimulus that subsequently influences the ability of the cell to respond to a second stimulus, which is important in pathogen defense and inflammation (156, 157). *In vitro*, CCL2 can prime monocytes and macrophages, thereby influencing the magnitude of the response to pathogenic immune triggers. Murine BMDMs (matured in L-cell-conditioned medium) that were pretreated with recombinant, endotoxin-tested CCL2 before stimulation with both IFNγ and LPS (classical activation stimuli) resulted in increased expression of iNOS (2-fold increase) and TNFα (4-fold increase) mRNA compared to cells that have not undergone CCL2 pretreatment (13). The underlying mechanism for the enhancement of classical activation is that CCL2 increased expression of miR9, which led to a decrease in the phosphatase Dusp6, a negative regulator of ERK signal transduction, which in turn impacts on gene expression of proinflammatory cytokines (13). Stimulation of PEMs (elicited with mineral oil or complete Freund's adjuvant) with LPS and recombinant CCL2 dose dependently decreased IL-12 production compared to cells treated only with LPS (109). In human monocytes, IL-12p70 was downregulated in response to stimulation with *Staphylococcus aureus* Cowan strain 1 (SAC) and IFNγ after preincubation with recombinant endotoxin-tested CCL2, whereas IL-10, TNFα, and TGFß1 were unaffected in this setup, and the extent of the response was donor dependent (83). Another study found that CCL2 synergistically enhanced LPS-induced M1 polarization in murine BMDMs. As this effect was diminished by Fasudil, a Rho-kinase inhibitor, the authors proposed a signaling cascade via Rho (99). To identify if CCL2 costimulation can influence cytokine profiles, another group cultured human GM-CSF differentiated macrophages with LPS and CCL2 compared to LPS alone and found that IL-10 was increased, and IL-6, IL-12p40, and TNFα protein levels stayed the same (100).

All the studies discussed above rely on adding recombinant CCL2. In the cases where endotoxin testing was performed on chemokine preps, any effect from contaminating LPS can be ruled out, although a CCL2-specific effect may not always be assumed without the use of CCL2-specific antagonism. However, there are data that corroborate these effects using different experimental setups that exclude recombinant protein contamination. For example, in a transgenic model, where human CCL2 was expressed in type II alveolar epithelial cells, it enhanced inflammatory response and led to a more activated appearance of monocytes upon i.p. injection of LPS or intravenous (i.v.) injection of yeast wall glucan compared to control mice, suggesting a sensitizing effect (141). Macrophages isolated from type 2 granuloma in mice treated with anti-CCL2 antibody showed significantly increased IL-12 production compared to control antibody (109). Isolated GM-CSF differentiated BMDMs from wild type and CCR2 knockout mice that were stimulated with LPS showed no difference in IL-10 protein levels but an increase in IL-6, TNFα, and CCL2 upon CCR2 knockout (100). A similar setup was investigated by a different group who found that, upon LPS stimulation of BMDMs (generated with M-CSF), the expression of iNOS, IL-12, TNFα, and IL-10 was increased compared to untreated cells and was significantly different between wild-type and CCL2 and CCR2 knockout mice. BMDMs of CCL2 and CCR2 knockout mice expressed significantly more iNOS and IL-12 but less IL-10 in comparison to their wild-type counterparts. In addition, BMDMs of CCL2 knockout mice expressed more TNFα compared to wild type (108). Together, these two paragraphs indicate that CCL2 might play a role in “good” priming to boost pathogen defense.

In addition, unfavorable, adverse priming effects have been observed. In severe burn injury, it was observed that CCL2 converts resident mesenteric lymph node macrophages to M2 subsets, as CCL2 knockout mice did not show a shift toward M2 macrophages compared to wild-type mice. Moreover, treatment of CCL2 knockout mice with recombinant CCL2 recovered the M2 shift as seen by increased expression of the M2 markers CCL17 and CXCL13. The observed shift toward M2 phenotype decreased host antibacterial innate immunity against sepsis stemming from oral *Enterococcus faecalis* infection in mice (106). Another detrimental effect of CCL2 to pathogen defense has been observed in systemic inflammatory response syndrome (SIRS) in mice (modeled via acute pancreatitis). SIRS led to the production of CCL2, which enhanced M2 polarization, thereby impairing the resistance of SIRS mice to infectious complications. When PEMs were cultured with SIRS mouse sera, the M2 chemokine CCL17 was increased significantly compared to incubation with normal mouse sera, and mannose receptor mRNA was expressed. PEMs isolated from mice injected with SIRS sera showed that CCL17 expression was increased drastically upon SIRS serum injection, and the effect was reversed by additional injection of anti-CCL2 antibody. PEMs isolated from normal mice treated with recombinant CCL2 showed an increased CCL17 expression compared to control mice (107).

This section has shown that CCL2 can act as an accessory to prepare cells for responding to subsequent stimuli and enhance effects of other signaling molecules by combinatorial signaling, thereby having an important role in pathogen defense. A striking example of the pathophysiological impact of CCL2 on myeloid cells in human disease was shown by Flores-Villanueva et al. who discovered that patients with the CCL2 allele G in the CCL2-promotor-enhancing region show a higher likelihood that a *Mycobacterium tuberculosis* infection will progress to active pulmonary tuberculosis. Patients with the GG genotype have higher CCL2 and lower IL-12p40 plasma levels compared to patients with the AA genotype. This was pinned down *in vitro* to higher CCL2 and resulting lower IL-12p40 production by monocytes in response to *Mycobacterium tuberculosis* antigens of healthy donors with the GG genotype compared to donors with the AA genotype (85). An influence of CCL2 in combination with LPS on reducing IL-12 expression has also been shown in several *in vitro* and *in vivo* assays in this section. All together, these human and murine data provide robust evidence for a non-redundant role of CCL2 in myeloid cell priming toward infection through the use of CCL2-blocking antibodies, CCL2 knockout mice, and transgenic mice in *in vitro* and *in vivo* experiments.

### CCL2 Impacts Myeloid Cell Maturation and Differentiation

In addition to influencing monocyte/macrophage biology, CCL2 has also been shown to influence differentiation and maturation of monocytes into DCs (see Table 4) and MDSCs. DCs circulate in the blood and migrate into lymphoid tissue, skin, and mucosa, where they capture foreign material, migrate to lymphoid organs, and present it to T cells. Thereby, they are able to transmit information to the adaptive immune system (158). DCs differentiated in the presence of CCL2, GM-CSF, and IL-4 displayed a markedly reduced production of IL-12 in response to CD40L and IFNγ compared to cells differentiated with GM-CSF and IL-4 alone and subsequent treatment with CD40L and IFNγ (118). This IL-12 suppression was in line with other studies of dexamethasone-treated human DCs (159) and LPS-treated, human immature DCs (117), where blocking CCL2 using a monoclonal antibody increased IL-12 production. Moreover, DCs matured in the presence of CCL2 have been shown to stimulate CD4+ T cells to produce less IFNγ vs. CCL2 untreated DCs (118). This effect was only observed when adding CCL2 during DC maturation, but not when added at the time of CD40L stimulation (83, 118), which underlines that CCL2 influences differentiation of monocytes into DCs. More evidence for CCL2's impact on DC differentiation comes from coculture experiments. A coculture of the trophoblast cell line HTR8 and human immature DCs showed that blocking CCL2 using monoclonal antibodies resulted in altered differentiation of DCs, evidenced by a decrease in CD14 expression indicating loss of monocytic cell properties and an increase in CD1a expression indicating gain of dendritic cell characteristics (117). It is of note that the response of DCs to CCL2 is dependent on their maturation state, as immature BMDCs from CCL2 and CCR2 knockout mice produced less IL-6 upon LPS stimulation, whereas mature, LPS-treated BMDCs from CCL2 and CCR2 knockout mice produced less IL-10 and more TNFα than those from wild-type mice (108).

**Table 4:** Summary of CCL2's effects on dendritic cells.

**Effect on dendritic cells**	**Source of CCL2 or CCL2/CCR2 blocking/KO**	**Cells/model**	**Methods and results**	**Reference**
**MATURATION/DIFFERENTIATION/CYTOKINE PRODUCTION**				
Monocyte to dendritic cell differentiation	Anti-CCL2 Ab	HTR8-DC coculture system during the differentiation of DCs +/– anti-CCL2 Ab	Anti-CCL2 Ab: monocytic marker CD14↓, DC markers CD1a↑, DC-SIGN (=) (FC)	(117)
Cytokine production	Anti-CCL2 Ab	HTR8-DC coculture system +/– anti-CCL2 Ab, stimulated with LPS	Anti-CCL2 Ab: IL-10↓, IL12p70↑ (ELISA)	(117)
	rCCL2	h CD14+ differentiated with GM-CSF + IL-4, primed +/– CCL2, then CD40L +/– IFNγ added; SNs analyzed	IL-12p70↓(ELISA), IL-12p35↓, IL-12p40↓(RT-PCR)	(118)
	rCCL2	h CD14+ differentiated with GM-CSF + IL-4, pretreated with CCL2 then combined TLR4/TLR8 activation; SNs analyzed	IL12↓ (ELISA)	(21)
	rCCL2	h DCs + CD4 cells cocultured; SNs analyzed	IFNγ of CD4+ cells↓ (ELISA)	(118)
	CCL2 KO, CCR2 KO	m BMDCs differentiated with GM-CSF, activated with LPS	Immature DCs: CCL2 KO and CCR2 KO: IL-6↓, mature DCs: CCL2 KO: IL-6↓, IL10↓, TNFα↑, CCR2 KO: IL10↓, TNFα↑ (ELISA)	(108)
Downregulates HLA-DR, upregulates PD-L1	rCCL2	h CD14+ cells differentiated with GM-CSF +/– CCL2 +/– LCN2	Costimulatory molecule HLA-DR↓, immune-suppressive molecule PD-L1↑ (FC)	(119)
**ENDOCYTOSIS**				
Increases endocytosis	CCL2 KO, CCR2 KO	m BMDCs differentiated with GM-CSF from wt and KO mice	KO mice: endocytosis↓ (Dextran endocytosis assay)	(108)

*LCN2, lipocalin-2; BMDC, bone-marrow-derived dendritic cell; SN, supernatant. ↑, upregulation; ↓, downregulation; (=), level stays the same*.

Myeloid-derived suppressor cells (MDSCs) are derived from granulocytic precursors, forming polymorphonuclear MDSCs (PMN-MDSCs) or from monocytic precursors, forming monocytic MDSCs, and arise in pathological conditions, such as chronic inflammation or cancer. They are renowned for their function to suppress T-cell responses and to support tumorigenesis (160). CCL2 has been shown to affect the differentiation of myelomonocytic cells into monocytic MDSCs. In an *in vitro* coculture system, CCL2 secreted from the trophoblast cell line HTR8/SVneo was shown to mediate the differentiation of CD14+ myelomonocytic cells into CD14+HLA-DR(-/low) MDSCs, as addition of anti-CCL2 antibody markedly reduced CD14 expression compared to untreated and isotype-control-treated cells. The effect on differentiation occurred at least partly in a STAT3-dependent manner (161). Another study by Fujisaka et al. indirectly implies an effect of CCL2 on MDSC differentiation. Hox antisense intergenic RNA overexpression in hepatocellular carcinoma cell lines Li-7 and Hep3B led to an increase in CCL2 expression in these cells. Subsequent coculture of these cells with peripheral blood mononuclear cells (PBMCs) led to PBMC differentiation to CD14+HLA-DR–CD33+CD11b+ MDSCs (162). However, abolishing this effect, for example, using anti-CCL2 blocking antibodies, was not tested.

CCL2 is also involved in the formation of osteoclasts (see Table 3). Osteoclasts are multinucleated cells formed via the fusion of mononuclear progenitors of the monocyte/macrophage lineage, which play a major role in bone resorption. Together with bone formation by osteoblasts, this process is crucial for the strength and integrity of the skeleton (163). The CCL2/CCR2 axis is involved in recruiting monocytes and preosteoclasts to remodeling sites, and several groups have shown that this axis is crucial for osteoclastogenesis, the formation of osteoclasts (102, 164–166). Along these lines, the CCL2/CCR2 axis is also required for the formation of foreign body giant cells (FBGCs), which can arise in the presence of foreign bodies, such as implants (102, 167). Absence of CCL2 or CCR2 caused by respective knockouts in bone marrow cells caused significant reduction in osteoclast and FBGC formation compared to wild-type counterparts (102). The importance of the chemokine receptor pair was also confirmed via blocking the CCL2/CCR2 axis with a dominant negative CCL2 mutant (7ND) *in vitro*, which led to inhibition of differentiation of colony forming unit-granulocyte macrophages (CFU-GM) into human osteoclasts by RANKL and M-CSF treatment vs. CFU-GMs not treated with 7ND (103). Furthermore, CCR2 and CCL2 knockout in mice were shown to result in elevated bone mass, visible in trabecular femur bone mineral density, trabecular bone volume, and trabecular number (104). Another publication by Binder et al. also showed that CCR2 knockout mice exhibit increased bone mass as observed in increased bone volume fraction, trabecular thickness, and bone mineral density, as well as decreased trabecular separation. All measured osteoblast markers (osteoblast-covered bone surface, calcein labeling, mineral apposition, osteoclastin concentrations) remained unaffected by CCR2 knockout compared to wild type, but osteoclast markers (osteoclast surface to bone ratio, osteoclast size, osteoclast nuclearity) were significantly reduced in these animals compared to wild type, which confirmed the effect on osteoclasts rather than osteoblasts (105). Further experiments showed that CCL2 signaling via CCR2 in murine osteoclasts *in vitro* resulted in activation of the ERK1/2 pathway. Addition of rCCL2 to wild-type osteoclasts treated with ERK1/2 inhibitor PD98059 led to decreased mRNA expression of Tnfrsf11a (RANK), an osteoclast marker, compared to CCL2 untreated cells. Induction of RANK expression by rCCL2 was almost completely abolished by CCR2 knockout. Moreover, knockout of CCR2 led to reduced nuclear factor kappa B (NF-κB) activation *in vitro* in murine osteoclasts compared to wild type, as measured by a NF-κB p65 DNA-binding assay. Last but not the least, CCR2 knockout mice were protected from osteoporosis caused by estrogen deficiency in a murine ovarectomy model compared to the wild-type ovarectomy group (105). The significance of CCL2/CCR2 signaling in osteoclast function is mirrored by its contribution to diseases of the bone, such as RA, osteoporosis, multiple myeloma, tooth eruption, and bone metastasis (102).

Summarizing, CCL2 was shown to be able to influence monocytes during their differentiation into DCs, MDSCs, and osteoclasts. Regarding the impact of CCL2 on the differentiation into DCs and MDSCs, *in vitro* data are available, in which the role of CCL2 was confirmed using CCL2-blocking antibodies. However, this function remains to be confirmed *in vivo*. For CCL2's role in osteoclast formation, plenty of *in vitro* data were obtained using anti-CCL2 antibody, a CCL2 mutant protein as well as recombinant CCL2, and data using CCL2 and CCR2 knockout mice were obtained, providing evidence for a non-redundant role of the chemokine and showing that the effect is mediated via CCR2. To further strengthen the data on this function, CCL2-blocking antibodies could be tested *in vivo*.

### Considerations Regarding CCL2's Impact on Myeloid Cells

A number of intriguing questions arise in the wake of work focusing on CCL2's influence on myeloid cells beyond chemotaxis. One of these questions centers around data showing downregulation of CCR2 expression of monocytes when differentiating into macrophages. CCR2 downregulation upon differentiation was reported by Denholm et al. for PMA-treated THP-1 cells (168) or human monocytes differentiated via culturing them *in vitro* for 7 days (169). There are also examples in the literature showing that macrophages respond very weakly to CCL2 in a chemotaxis assay (170). Thus, do macrophages even express the receptor that would enable them to respond to CCL2? Sierra-Filardi et al. performed profiling of human and murine macrophages by reverse transcription PCR (RT-PCR) and fluorescence-activated cell sorting and showed that GM-CSF differentiated macrophages do express CCR2 (100). Moreover, evidence from experiments using macrophage cell lines *in vitro* (summarized in Table 3) show an effect of CCL2 treatment and CCL2 blocking on macrophage polarization (95, 98, 99, 101) or anti-CCL2 treatment (101). Much evidence showing that macrophages can respond to CCL2 also comes from studies done on osteoclasts, which are tissue-resident macrophages (102–105). Concluding, it seems that macrophages represent a wide spectrum of differently polarized cells wherein some subsets do express CCR2 and are able to respond to CCL2 while others do not (171–173). Regulation of cell expression of this chemokine receptor may represent a neat way of controlling site-specific macrophage responses to CCL2.

Another important caveat to consider is the potential for endotoxin contamination in recombinant CCL2 protein preparations used for *in vitro* and *in vivo* studies to contribute to monocyte/macrophage activation. Only very few of the above reviewed papers provide information about the endotoxin level of the recombinant CCL2 used in the studies. A large number of papers mentioned the supplier, whereas some did not mention the source of recombinant CCL2 at all. It will be important not only to test LPS levels but also to include anti-CCL2 antibodies to show specificity going forward when examining cell activation in this way.

An important fact that is often overlooked is binding of CCL2 to its coreceptors, glycosaminoglycans and proteoglycans, as this can influence the chemokine's oligomerization state, presentation, and, therefore, function (59, 174–176). Apart from heparan sulfate proteoglycans, the CCL2/CCR2 axis was found to be involved in versican's (chondroitin sulfate proteoglycan) metastasis promoting role in murine and human models of diseases (177), and versican was shown to protect CCL2 from degradation and to enhance its chemotactic properties (178). These molecular aspects are rarely taken into consideration when investigating CCL2's functional roles, as the recombinant chemokine is usually added in solution, whereas *in vivo*, it is most likely presented by components of the extracellular matrix. This might help to explain its varying, context-dependent functions.

Moreover, when interpreting the outcomes of different experiments, it is essential to consider whether CCL2 or CCR2 was targeted. CCR2 can be activated by other ligands than CCL2, namely, CCL7, CCL8, CCL12 (mouse only), CCL13, CCL16, and the chemoattractant PC3-secreted microprotein (179–181), and its manipulation has wider ranging effects than targeting CCL2 alone, which might be compensated by the upregulation of other CCR2 ligands. In addition, expression profiling of wild-type, CCL2 knockout, and CCR2 knockout murine monocytes showed alterations in multiple genes between the different genotypes, especially in those that are involved in development and function (182). This may be responsible for diverging responses in CCL2 knockout vs. CCR2 knockout. In addition, cellular responses following activation of CCR2 can be cell-type specific due to coupling of the receptor to different G-proteins (183, 184).

Temporal aspects of CCL2 stimulation also seem to be very important on the functional outcome of stimulation (13). In knockout mice, the chemokine receptor is constantly knocked out, whereas antibodies or recombinant protein can be administered only at certain timepoints (182). Furthermore, in knockout animals, CCL2 and CCR2 are usually knocked out systemically and not in a tissue- or cell-type specific manner, which results in far reaching systemic effects. CCL2's function of attracting monocytes is indispensable to get monocytes to the center of inflammation (142, 143) or cancer (16), and therefore, this property is a prerequisite for being able to exert other effects on those cells. Therefore, knockout mouse models may not be suitable to answer all questions regarding CCL2's functions on myeloid cells, and alternative setups, for instance using CCL2-blocking antibodies, should be considered. Finally, some effects might occur only in a defined range of CCL2 concentrations (13).

Another interesting fact to consider is that CCL2 induces expression of MCP-1-induced protein (MCPIP, Regnase-1, Zc3h12a) (185), which was found to promote an M2 phenotype in macrophages by inhibition NF-κB activation, sequential induction of ROS, ER stress, and autophagy, and induction of C/EBPbeta and PPARy (186). Furthermore, it was shown that MCPIP/Regnase-1 is responsible for degradation of IL-6 and IL-12p40 mRNA in macrophages (187). Additional effects of MCPIP/Regnase-1 in controlling immune responses have been reviewed in Takeuchi (188). This raises the possibility that some of the effects may not be directly caused by CCL2, but via CCL2-induced factors.

Altogether, while new data are starting to reveal a role for CCL2 beyond chemoattraction, more detailed *in vitro*, mechanistic, and *in vivo* studies are required to fully understand CCL2's position among myeloid cell activating, polarizing, and priming stimuli.

### CCL2 Enhances Myeloid Cell Survival and Proliferation

Looking beyond myeloid cell activation and differentiation, a number of studies have examined the impact of CCL2 on the survival of these innate immune cells. In human CD11b+ cells under serum starvation, addition of recombinant CCL2 upregulates antiapoptotic proteins cFLIPL, Bcl-2, and Bcl-XL and inhibits cleavage of caspases−3, −6, −7, −8, and lamin A, all of which contribute to promoting CD11b+ cell survival (10). In human PBMC-derived, M-CSF, and LPS-treated macrophages, CCL2 secretion is regulated via NFAT5, which is in turn regulated by proinflammatory M1 polarizing and hypoxic stimuli, and confers apoptotic resistance to human RA macrophages and murine peritoneal and splenic macrophages. This was shown in NFAT5-silencing experiments in which NFAT5-silenced human RA synovial fluid macrophages were incubated with or without CCL2 addition, and the apoptosis marker Annexin V was measured by flow cytometry. Furthermore, treatment with anti-CCL2 antibody increased apoptosis in macrophages from wild-type mice. Moreover, expression levels of NFAT5 and CCL2 were significantly higher in matched RA synovial fluid CD14+ cells (comprised mainly of macrophages) than in peripheral CD14+ cells, highlighting the potential importance of these molecules in this disease. However, the authors conclude that CCL2 alone is probably not entirely responsible for NFAT5-mediated RA macrophage survival, as NFAT5 regulates a number of genes involved in macrophage apoptosis (32).

CCL2 stimulated proliferation and polarization of human macrophages (generated with M-CSF) into myeloma-associated macrophages, a subset of bone marrow infiltrating cells in multiple myeloma that has been shown to be responsible for drug resistance (126, 189). Increased proliferation was observed for human macrophages incubated *in vitro* in the presence of CCL2 vs. untreated cells in an 3-(4,5-Dimethylthiazol-2-yl)-5-(3-carboxymethoxyphenyl)-2-(4-sulfophenyl)-2H-tetrazolium (MTS) assay, which is a colorimetric assay based on the conversion of the tetrazolium dye MTS to a formazan by mitochondrial enzymes (190). Moreover, CCL2 induced expression of myeloma-associated macrophage markers IL-6 and c-myc when added to the media, compared to CCL2-untreated cells. This effect was achieved by activating growth and survival signaling via PI3K/Akt and ERK/MAPK pathways, as addition of PI3K-Akt inhibitor LY294002 or Erk1/2 inhibitor U0126 to the cell media of myeloid-associated macrophages inhibited cell proliferation vs. untreated cells (126). Another different action on macrophage survival has been observed by treatment of macrophages with CCL2, which induced macrophage cell division in adipose tissue explants measured by a 5-ethynyl-2′-deoxyuridine (EdU) incorporation assay. Furthermore, *in vivo* CCL2 knockout decreased macrophage proliferation in adipose tissue of high-fat diet compared to wild-type mice also measured by EdU incorporation (112).

In this context, the question arises whether macrophages, which have long been regarded as terminally differentiated immune cells, are actually able to proliferate. The MTS assay that Li et al. were using is an assay determining metabolic activity of cells used as proxy for cell viability/proliferation and shows a decrease (when blocking CCL2) or increase (when adding CCL2) in signal intensity compared to control. If this is not due to proliferation, the other option would be that the observed effect is due to viability. The authors further showed that PI3K-Akt and MAPK/Erk pathways were activated, which not only promote cell proliferation but could also improve survival. The EdU incorporation assay performed by Amano et al. however, is an assay specifically designed for detecting cell proliferation by EdU incorporation of cells during the S-phase and clearly showed a positive signal. Furthermore, there are publications showing that macrophages can self-renew as reviewed in Jenkins et al. (191) and Sieweke and Allen (192). Independent of the underlying process (survival or proliferation), it can be concluded that more viable or more metabolically active macrophages were detected upon CCL2 treatment.

Neutrophils defend the host against pathogen infection via phagocytosis, degranulation, and generation of neutrophil extracellular traps and the release of a variety of effector molecules (193). In neutrophils, addition of soluble CCL2 induced the activation of PI3K/Akt, ERK, and NF-κB *in vitro*, which led to a decrease in constitutive apoptosis in these cells. CCL2 exerted this antiapoptotic effect in a CCR2-dependent manner as tested using the CCR2 receptor antagonist RS102895 and an anti-CCR2 antibody (194). Interestingly, IFNγ, which is known for its antiapoptotic effect on neutrophils, and which does not affect CCL2 synthesis alone, can cause expression and secretion of CCL2 in the presence of LPS in human neutrophils *in vitro* (25). Moreover, GM-CSF, which also has been shown to be an antiapoptotic cytokine for neutrophils (195) induces secretion of CCL2 in human neutrophils *in vitro* (196). Whether CCL2 expression is causative for the observed antiapoptotic effects of IFNγ and GM-CSF on neutrophils or merely acts as a messenger has not been shown, but might be an interesting hypothesis to test.

Together, these data indicate that CCL2 not only recruits immune cells to tissues but may also prolong their residence at the sites of inflammation by impacting on apoptosis and/or survival-controlling molecules. The majority of data summarized in this section has been obtained using *in vitro* assays. The use of anti-CCL2 antibodies and a CCR2 receptor antagonist and anti-CCR2 antibody point toward a CCL2-specific effect mediated via CCR2. Apart from one experiment by Amano et al. using CCL2 knockout mice (112), more conclusive evidence from *in vivo* studies using knockout mice or blocking antibodies is warranted.

### CCL2 Enhances Host Defense, Cellular Cleanup, and Allergic Responses

In addition to the effects on myeloid cell migration, phenotype, and survival, CCL2 may also impact further, diverse and far ranging immune cell functions that are summarized below.

#### Increase of Phagocytosis, Efferocytosis, and Endocytosis of Macrophages

Macrophage efferocytosis (recognition and engulfment) of apoptotic cells has been shown to be increased by CCL2. Upon treatment with recombinant CCL2, murine alveolar macrophages, PEMs, and the J774 macrophage cell line exhibited increased efferocytotic activity. This was shown *in vitro* using an assay in which macrophages were cocultured with apoptotic human neutrophils after treatment, with or without CCL2, and staining with Diff Quik to visualize ingested apoptotic cells. This was confirmed in a murine *in vivo* model where apoptotic human neutrophils were injected into the lungs of mice treated with CCL2 and staining lavaged alveolar macrophages with MPO for neutrophil apoptotic bodies, vs. CCL2-untreated control mice. This was mediated in a Rac1/PI3-kinase-dependent manner (110). When investigating BMDMs (M-CSF derived) and BMDCs (GM-CSF derived) generated from CCL2 knockout and CCR2 knockout mice, Chen et al. found reduced phagocytosis (of *Escherichia coli*) and endocytosis (of dextran) compared to BMDMs of wild-type mice (108). Furthermore, the CCL2/CCR2 axis seems to be essential for macrophage-mediated endocytosis of collagen and fibrin (111, 171). This ECM turnover is critical during tissue remodeling and repair (197).

#### Activation of Autophagy of Monocytes

Autophagy is a constitutive, cellular mechanism of degradation of damaged proteins and cellular organelles. It can be enhanced under various physiological and pathophysiological stimuli, such as inflammation, hypoxia, and starvation (198). A hyperactivation of autophagy was observed in human CD11b+ cells upon CCL2 treatment (10). This was shown by detecting an increase in the LC3-II protein (resulting from cleavage off the microtubule-associated protein LC3) that correlates with autophagosome number (199). Together with their other findings of CCL2's influence on cytokine production and macrophage polarization (see *CCL2 Induces Context-Specific Macrophage Polarization and Cytokine Secretion*), Roca et al. suggest that autophagy might play a role in M2 macrophage polarization (10).

#### Induction of Respiratory Burst in Monocytes

Respiratory burst is an essential line of host defense characterized by increased oxygen uptake into phagocytic cells and subsequent production of large quantities of microbicidal reactive oxygen species (200). In human monocytes, CCL2 was shown to induce respiratory burst (91), and a CCL2-induced N-acetyl-β-glucosaminidase and superoxide anion release has been described (92). These effects have not been confirmed in *in vivo* models yet.

#### Activation of Mast Cells

Mast cells can be found in connective tissues throughout the body. They are rich in granules containing inflammatory mediators (e.g., eicosanoids, histamine) and contribute to inflammatory responses, such as allergic reactions (201). CCL2 is a well-established activator of mast cells. The chemokine has been shown to activate murine mast cells and led to mast cell degranulation *in vitro* and *in vivo* (202–204). *In vitro*, it caused profound histamine release from rat mast cells (205, 206), as well as release of serotonin (rat) (205) and the leukotriene C4 (LTC4) (mouse) (203). Furthermore, it induced mast cell aggregation and cytoplasmic communication between rat mast cells *in vitro* (205). The effect of CCL2 on mast cells in allergic reactions has been shown in a mouse model of ragweed-pollen-induced allergic conjunctivitis, where blocking of CCL2 or its receptor CCR2 by antibodies suppressed clinical signs of hyperreactivity and mast cell degranulation in the animals (204). Similar findings were reported in a cockroach-allergen-induced mouse model of allergic bronchial hyperreactivity, where anti-CCL2 antibodies and CCR2 knockout were able to attenuate the allergic reactions (203). Therefore, controlling CCL2 levels in allergic diseases might prove to be useful in the clinic.

#### Activation of Basophils and Eosinophils

Granulocytes are characterized by the presence of granules in their cytoplasm and are subdivided in neutrophils, eosinophils, and basophils. After maturation and proliferation in the peripheral circulation, eosinophils exert their function in tissues, where they release cytotoxic granule proteins, lipid mediators, cytokines, and other proinflammatory substances. They play a role in parasitic defense, as well as in allergies and asthma (207). Basophils, like mast cells, modulate inflammatory responses by releasing histamine and a variety of cytokines to recruit various effector cells (Th2, macrophages, and others) to sites of inflammation (208). *In vitro*, CCL2 induced release of histamine from human basophils (209, 210) as well as release of sulfido-leukotrienes (211) and promoted LTC4 production in IL-3, IL-5, and GM-CSF-pretreated human basophils (212). These degranulating effects of CCL2 were accompanied by profound structural changes of basophils, such as granule-vesicle attachments, piecemeal degranulation, anaphylactic degranulation, and uropod formation that differ quantitatively from other secretagogue agents (213).

The impact of CCL2 on eosinophils is less clear. The chemokine does not directly attract these cells (214, 215). However, studies suggest that it might serve as an indirect mediator of eosinophil activation and attraction. In a mouse model of ragweed-pollen-induced allergic conjunctivitis, treatment with an anti-CCL2 Ab inhibited eosinophilic recruitment (216). In another study, SiglecF+/CD11c– eosinophils from chitin challenged CCR2 knockout mice expressed markedly less CCL5, IL-13, and CCL11 than respective cells from wild-type mice (101). These observations may be explained by a disruption of CCL2-dependent attraction of T lymphocytes to sites of allergic inflammation and subsequently missing attraction and activation of eosinophils by the intricate interplay between αβ T lymphocytes and γδ T lymphocytes that recruits eosinophils, which was reviewed by de Henriques and Penido (217).

#### CCL2 Plays a Role in Lipid Body Biogenesis and Instrumentation in Macrophages

Lipid bodies (lipid droplets) are cellular organelles occurring in large amounts not only in adipose tissue but also in non-adipose tissue, which are an important part of lipid metabolism. More recently, lipid bodies have been shown to also be involved in processes such as endoplasmatic reticulum stress and oxidative stress as well as protein storage and turnover (218). CCL2 plays a role in equipping newly formed lipid bodies with leukotriene B_4_ synthetic function (115). Pacheco et al. discovered *in vitro* that the process was mediated via a CCR2-, ERK1/2-, PI3K-, and cytoskeleton-dependent mechanisms (115). Later, the role of the CCL2/CCR2/ERK axis in oxidized low-density lipoprotein (oxLDL) induced lipid body biogenesis has been confirmed, and the findings were extended by showing that anti-CCL2-Ab pretreatment inhibited the expression of adipose differentiation-related protein upon oxLDL stimulation in macrophages (116). The pivotal role of CCL2 in the process was confirmed *in vivo* in sepsis (cecum ligation and puncture) and endotoxemia models of inflammation, where CCL2 knockout led to a decreased lipid body number per cell and decreased amount of leukotriene B_4_ (115). Moreover, further data regarding the reduction in lipid body formation upon anti-CCL2-Ab treatment *in vitro* and the dependence on CCR2 signaling *in vivo* were shown (116). This mechanism is part of innate immune responses to infections and inflammatory processes and is involved in the formation of atherosclerosis (116).

All together, these data reveal not only surprisingly wide-ranging target cell types that respond to CCL2 activation but also implicate a multitude of effector functions resulting from this interaction, from cellular cleanup to cellular metabolism and allergic responses.

### CCL2 Can Set Up Effector Molecule Feedback Loops in Tumor-Immune Cell Crosstalk

Multiple studies describe an important role of CCL2 in the tumor microenvironment in different types of cancer. Apart from directly influencing tumor and stromal cells (74, 76, 77) and attracting immune cells into the tumor microenvironment (69), more recently, an additional role in mediating tumor-immune cell crosstalk has been ascribed to CCL2 (see Table 5). As described in *CCL2 Induces Context-Specific Macrophage Polarization and Cytokine Secretion* of this review, macrophages polarize in response to cues of their microenvironment. In the context of cancer, it is worth noting that M1 classically activated macrophages possess antitumorigenic properties, whereas M2 alternatively activated macrophages are ascribed protumorigenic properties (219).

**Table 5:** Summary of CCL2's effects on macrophages in cancer.

**Effect on macrophages**	**Source of CCL2 or CCL2/CCR2 blocking/KO**	**Cells/model**	**Methods and results**	**Tumor type**	**References**
**MATURATION/DIFFERENTIATION/CYTOKINE PRODUCTION**					
Promotes M2 phenotype	Anti-CCL2 Ab + Anti-CCL12 Ab	m flank tumor model +/– blocking Abs	Blocking Abs: M2 marker CD206↓ (FC), Blocking Abs: M1 marker iNOS↑, M2 markers FcR1↓, CD206↓, Arg1↓ (RT-PCR)	NSCLC	(120)
	SN of tumor cells, rCCL2	Coculture of RAW264.7 macrophages and 4T1 m breast cancer cells	M2 marker CD206↑ (FC)	Breast cancer	(121)
	CM of tumor cells, CCR2 antagonist, anti-CCL2 Ab	m BMDM and CM of Hepa1-6 cells	M2 markers Arg1↑, IL-10↑, M1 markers iNOS↓, IL12↓, immune suppressive cytokines: G-CSF↑, MIP-1↑, MIP-2↑, CCL2 blocking or CCR2 antagonist: effects reversed (RT-PCR)	Hepatocellular carcinoma	(122)
	SACC-83 (adenoid cystic carcinoma cell line) CM, CCR2 antagonist RS504393	Macrophages derived from THP-1 cells treated with PMA +/– RS504393 + CM, murine SACC-83 xenograft model +/- RS504393	Macrophages with SACC-83 CM: M2 marker CD163↑, CD206↑ (FC), M2 marker Arg1↑, IL-10↑, M1 marker TNFα↓, IL-1β↓ (RT-PCR), GDNF expression↑ (RT-PCR, WB, ELISA), in presence of RS504393: M1 markers Arg1 and IL-10↓ (RT-PCR), murine SACC-83 xenograft model + RS504393: tumor associated M2 macrophages CD163↓ (immunohistochemical cell counts)	Adenoid cystic carcinoma	(123)
Cytokine production	CCR2 antagonist	m flank tumor model +/– CCR2 antagonist	CCR2 antagonist: immune suppressive cytokines↓ (cytokine profiling of sorted (F4/80, CD11b, CD206) macrophages)	Hepatocellular carcinoma	(122)
	Anti-CCL2 Ab	TAM isolated from mammary tumors +/– anti-CCL2 Ab	blocking CCL2: IL-1β↓, marker genes (=) (Arg1, CD206, decoy IL1R2, iNOS), inducers of IL-17 (=) [TGFβ, IL-6, Il23p19)] (RT-PCR)	Breast cancer	(124)
	SN of tumor cells (+/– transfected with antisense CCL2), rCCL2	PEMs (activated and resting) or isolated TAM + 4T1 cell SNs or rCCL2	PEMs + tumor-derived CCL2: proinflammatory cytokines TNFα ↑, IL18↑, IL-12 not detectable (RT-PCR), rCCL2 treatment of PEMs: IL12↑ (ELISA), TAM from tumors +/– CCL2: IL12 (=), TNFα (=), IL18 (=) (ELISA)	Breast cancer	(125)
Proliferation and polarization	rCCL2, anti-CCL2 Ab, PI3K-Akt inhibitor, Erk1/2 inhibitor	h macrophages stimulated with M-CSF +/– cocultured in Transwell system with ARP-1 or MM.1S MM cells to obtain MM-associated macrophages	Blocking CCL2: proliferation↓, adding rCCL2: proliferation↑, blocking PI3K-Akt and ERK1/2: proliferation↓ (MTS assay) adding rCCL2: phosphorylation of different kinases↑, IL-6 (=), c-myc↑, cyclin D1↑, p27Kip1↓ (WB)	Multiple myeloma	(126)
**ACTIVATES KILLING**					
Primes macrophages to become tumoricidal together with LPS	Tumor cells transfected +/– CCL2, tumor cell SN, Anti-CCL2-Ab	PEMs (+/–LPS) and CT26 tumor cells (+/–CCL2 transfected), +/– anti-CCL2 Ab	Cytotoxicity in presence of CCL2↑, blocking CCL2: cytotoxicity↓ (release of radioactivity from target cell DNA)	Colon carcinoma	(127)
	Tumor cells transfected +/– CCL2, rCCL2	PEMs (+/–LPS) and melanoma and fibrosarcoma tumor cells (syngeneic)	Cytotoxicity ↑(release of radioactivity from target cell DNA)	Melanoma	(128)
	SN of NIH3T3, Anti-CCL2 Ab, rCCL2	PEMs and RENCA cells + SN of CCL2-transfected NIH3T3 +/– LPS, +/– anti-CCL2-Ab or rCCL2, alveolar macrophages isolated +/– after injection of CCL2-transfected NIH3T3 and RENCA cells	Cytotoxicity in presence of CCL2 ↑, blocking of CCL2: cytotoxicity↓ (release of radioactivity from target cell DNA)	Renal adenocarcinoma	(129)
	*In vivo* model	Injection of adenocarcinoma cells transfected +/–CCL2 complementary DNA +/- LPS i.p.	CCL2 + LPS: tumor size↓ (measuring footpad/tumor size)	Adenocarcinoma	(130)
Increases macrophage-mediated cytotoxicity	rCCL2, MEK inhibitor PD98059	PEMs cells + P815 (m mastocytoma) cells + rCCL2 +/– PD98059, PEMs + rCCL2	Cytotoxicity with rCCL2↑, with PD98059↓ (release of radioactivity from target cell DNA), PEMs with rCCL2: Phospho-p42/44 MAPK↑, phospho-JNK↑, phospho-c-Jun↑ (WB)	Inflammation and tumor growth	(113)
No direct influence on macrophage-mediated anti-tumor activity	SN of tumor cells (+/– transfected with antisense CCL2)	PEMs (activated and resting) from female Balb/c mice + 4T1 tumor cell SNs	Phagocytic activity (=) (phagocytosis assay), cytotoxicity (=) (cytolytic activity assay), nitric oxide levels (=) (colorimetric detection)	Breast cancer	(125)

*Ab, antibody; BMDM, bone-marrow-derived macrophage; CM, conditioned medium; FACS, fluorescence-activated cell sorting; FC, flow cytometry; h, human; KO, knockout; m, murine; MM, multiple myeloma; NSCLC, non-small cell lung cancer; PEM, peritoneal exudate macrophage; RT-PCR, reverse transcription PCR; rCCL2, recombinant CCL2; SSC, side scatter; SN, supernatant; TAM, tumor-associated macrophage; WB, Western blot; ↑, upregulation; ↓, downregulation; (=), level stays the same*.

In a coculture model of RAW264.7 macrophages and 4T1 mouse breast cancer cells, CCL2 and VEGF-A promoted the formation of M2 macrophages, detected via the M2 marker CD206 (121). In another setup, monocytic-cell-derived TNFα upregulated CCL2 secretion from tumor cells, which then promoted TNFα secretion from monocytic cells, thereby forming a vicious circle of cross-regulation that may contribute to tumor progression and malignancy (84). Brault et al. demonstrated that tumor-derived CCL2 was able to modify cytokine gene expression (increased TNFα, IL-18), but not protein expression, in PEMs and tumor-associated macrophages (TAMs). Interestingly, they observed a difference in the effects of tumor-derived CCL2 and recombinant CCL2, being that only the latter was able to induce IL-12 protein expression. This may have been caused by different posttranslational modifications (PTMs) of CCL2 (discussed in *Can CCL2 Inhibit Tumor Cell Growth and Enhance Tumor Cell Killing by Myeloid Cells?*), the presence of other factors in the tumor cell supernatant that suppressed IL-12 production, or the presence of bacteria-derived contaminants (discussed in *Considerations Regarding CCL2's Impact on Myeloid Cells*), which can be present in recombinant protein preparations and can cause IL-12 stimulatory effects (220, 221). No information about the presence of LPS or lipoproteins could be found in the above paper (125).

More evidence also points toward an involvement of the CCL2/CCR2 axis in the crosstalk between tumor cells and macrophages leading to immunosuppression. In a cell-based assay, murine BMDMs were exposed to the conditioned media of murine hepatoma cell line Hepa1-6 cells to mimic microenvironmental interactions. After exposure of BMDMs to the conditioned medium, the expression of M2 marker genes, Arg1 and IL-10, were upregulated, and the effect could be blocked with a CCR2 antagonist or anti-CCL2 antibody. Furthermore, iNOS and IL-12 were upregulated upon blocking CCR2 or CCL2 (122). In human CD11b+ cells, IL-6 and CCL2 induced the expression of each other. Treatment of isolated human CD11b+ cells incubated with CCL2 increased IL-6 expression more than 5-fold compared to untreated cells, measured with cytokine arrays. Likewise, IL-6 treatment induced CCL2 expression 2-fold (10). In their publication, the authors suggested a mechanism by which CCL2 and IL-6 potentiate tumor progression, as the tumor infiltrating monocytes are protected from apoptosis (see *CCL2 Enhances Myeloid Cell Survival and Proliferation*) and skewed toward a protumorigenic M2 phenotype (10). On the contrary, in another study, IL-6 mRNA (measured by cytokine array) and protein levels (measured by ELISA) of human monocytes derived from PBMCs by CD14+ sorting and M-CSF treatment were upregulated in the presence of anti-CCL2 antibody vs. isotype control (100).

In a model performed by Kersten et al. BMDMs (differentiated with murine M-CSF, primed with LPS) were incubated *in vitro* in the presence of murine lobular breast cancer cell line KEP conditioned medium, which resulted in 2-fold IL-1β upregulation that returned to the control level upon CCL2 blockade. *In vivo*, TAMs from KEP tumors of CCL2-blocking antibody-treated mice showed significantly decreased IL-1β mRNA expression vs. control KEP tumor-bearing mice (124). These findings indicate that tumor-derived CCL2 initiates the expression of TAM-derived IL-1β. IL-1β, itself, has been previously shown to stimulate IL-17 production in γδ T cells (222), a subgroup of T cells characterized by the presence of γ and δ T cell receptor chains and non-MHC restricted antigen recognition (223). While IL-17 plays an important role in host defense (224), it has also been shown to enhance tumor progression by conferring T cell suppressive properties to neutrophils (222). In summary, CCL2 has the potential to drive via IL-1β the γδ T cell/IL17/neutrophil axis, which promotes breast cancer metastasis (124, 222).

In another study, the effect of tumor-derived CCL2 was investigated by incubating macrophages (derived from THP-1 cells by PMA treatment) with conditioned medium of the salivary adenoid cystic carcinoma cell line SACC-83. The authors showed that the tumor-cell-conditioned medium increased the percentage of M2 macrophages determined via RT-PCR and upregulated M2 polarization markers CD163, CD206, IL-10, and Arg1 while downregulating M1 marker TNFα. Moreover, tumor-derived-conditioned medium induced the expression of glial-cell-derived neurotrophic factor by TAMs on mRNA and protein level, which was involved in promoting proliferation, migration, and invasion of SACC cells. All these effects were reversed when the CCR2 antagonist RS504393 was added. Furthermore, *in vivo*, the CCR2 antagonist significantly reduced the number of CD163 M2 TAMs (123).

*In vivo*, the function of CCL2 in the tumor microenvironment was investigated using a CCL2-blocking antibody together with a CCL12-blocking antibody, as two CCL2 orthologs bind to CCR2 in the mouse, namely, murine CCL2 and CCL12 (120, 179). A change occurred in the polarization of TAMs to a more antitumoral phenotype as defined by a reduced percentage of tumor-associated M2 macrophages (CD11b+, Ly6G–, F4/80+, CD206+) after CCL2/CCL12 blockade in an animal flank tumor model in which mice were injected in the right flank with tumor cells and tumors were treated with anti-CCL2/anti-CCL12 antibodies or saline as control. Furthermore, mRNA levels of the M2 markers FcR-1, CD206, and Arg-1 were reduced to ~60% of control levels after treatment with the antibodies. This was associated with the activation of cytotoxic CD8+ T lymphocytes (CTLs) (120). Another group tested a CCR2 antagonist *in vivo* in a subcutaneous tumor model, where treatment led to decreased production of M2 cytokines and chemokines by TAMs (122). In patients with breast cancer, a positive correlation of CCL2 with IL-1β and with macrophage marker CD68 in all breast cancer types was shown (124). Furthermore, the relevance of these findings was also confirmed in human hepatocellular carcinoma tumor tissue stainings, where CCL2 expression in tumor cells correlated with the tumor-infiltrating CD68+ TAM and lower numbers of antitumorous CD8+ T cells (122).

Summarizing, this paragraph points toward a tumor-promoting, immunosuppressive role of CCL2 in the tumor microenvironment. By including CCL2 and/or CCR2 blocking antibodies in their *in vitro* setups several groups confirmed that the observed effect was indeed based on the CCL2/CCR2 axis. Moreover, the importance of CCR2 signaling was confirmed in a series of *in vivo* studies, using CCR2 antagonists and blocking antibodies against two of its ligands, namely, CCL2 and CCL12. The missing pieces in this puzzle are *in vivo* studies specifically targeting only CCL2.

### Can CCL2 Inhibit Tumor Cell Growth and Enhance Tumor Cell Killing by Myeloid Cells?

In contrast to *CCL2 Can Set Up Effector Molecule Feedback Loops in Tumor-Immune Cell Crosstalk*, where CCL2 is involved in promoting tumor growth via suppressing the immune response, a series of older papers demonstrate an inhibitory effect on tumor cell growth of CCL2. Stimulation of human monocytes with CCL2 in a study by Matsushima et al. led to a growth inhibitory effect for the HT29 colon cancer cell line and the A375 C-5 melanoma cell lines assessed with a [^3^H]-thymidine incorporation assay *in vitro*, in which monocytes were grown adherently and tumor cells were added in suspension for coculturing and the incorporation of [^3^H]-thymidine was measured (37). Similar findings pointing toward a tumoricidal effect of CCL2 have been shown by Rollins et al. who injected CCL2-expressing and CCL2-non-expressing Chinese hamster ovary cells into nude mice, compared their tumor-forming potential, and found that CCL2-expressing cells were not able to form tumors *in vivo*, whereas CCL2-non-expressing cells did form tumors. The authors did not confirm whether the effect was mediated by monocytes. However, this seems plausible, as mostly monocytes were attracted to the site of tumor cell injection (93). Asano et al. used human brain tumor cells with different CCL2 expression levels (native HBT28: high CCL2; HBT20: low CCL2 and CCL2-transfected HBT28 and HBT20 cells) and showed that the propensity of LPS-activated human monocytes to inhibit tumor cell growth was dependent on the basal CCL2 expression level of the tumor cells (94). These three papers suggest an interplay between CCL2 and monocytes that leads to decreased tumor cell growth.

More evidence shows that CCL2 mediates tumor cell killing indirectly by activating macrophages. The macrophage-activating effects of CCL2 were shown as treatment with CCL2 induced phosphorylation of p42/44 MAPK in murine peritoneal macrophages *in vitro*, which could be blocked by pretreatment with MEK inhibitor PD98059. Moreover, CCL2 treatment induced transcription and phosphorylation of the transcription factor c-Jun (113). Furthermore, the authors could show that CCL2 treatment led to a drastic change in actin redistribution, bundling, and aggregation, which is a typical response of leukocytes to chemokines [as reviewed in (225)] and could also be blocked by PD98059. The functional consequences of their findings were shown in a cytotoxicity assay, where CCL2 treatment enhanced macrophage-mediated cytotoxicity and accompanying TNFα expression. The effect could be inhibited by PD98059 in a dose-dependent manner. Altogether, they showed that CCL2 is an activator of PEMs via the MAPKs ERK1, ERK2, and JNK (113). Cytotoxicity of macrophages is modulated via CCL2 by increasing the level of membrane-bound FasL, a protein involved in apoptosis, which renders CCL2-primed macrophages more cytotoxic. CCL2-primed macrophages (RAW264.7) killed primary smooth muscle cells (SMCs) through a FasL/Fas-Caspase8-RIP1-mediated mechanism, and cell–cell contacts were required for the effect, as conditioned medium of primed macrophages added to SMCs did not achieve the same effect as adding the primed cells (98).

A series of publications suggest that CCL2 can also synergize with bacterial endotoxins to activate macrophages to become tumoricidal. In one assay, PEMs were cultured in the presence of LPS and supernatant of CT26 (murine colon carcinoma) tumor cells. Subsequent coculturing of these macrophages with CT26 cells showed that macrophage cytotoxicity (assessed by [3H]-thymidine release from lysed cells) toward CT26 tumor cells was increased in a CCL2-dependent manner, as the addition of CCL2 strongly increased cytotoxicity and the addition of anti-CCL2 antibody reduced cytotoxicity. The findings were confirmed *in vivo*, where CCL2-positive tumor cells produced significantly fewer lung metastases (127). Along this line, various tumor cells transfected to express CCL2 were significantly lysed by PEMs treated with LPS, whereas parental (untransfected) or control cells (transfected with control complementary DNA) were not. In addition, when using the bacterial products lipopeptide and muramyl tripeptide phosphatidylethanolamine to activate PEMs *in vitro*, the effect on cytotoxicity was very similar (128).

Singh et al. showed that the combination of CCL2 and LPS acted synergistically in activating macrophages, as the capacity of each of the stimuli alone to activate the tumoricidal properties of macrophages was significantly lower. Owing to the fact that, in their assays, the sequence of the stimuli was important (first chemokine stimulation, then LPS stimulation), the authors suggested that CCL2 can prime macrophages to respond to a subsequent signal, which corresponds well with CCL2's ability to prime myeloid cells described in *CCL2 Primes Cells to Respond to Subsequent Infection* of this review (128). Similar findings were also obtained in an *in vivo* model, where tumor cells producing high levels of CCL2 (transfected with CCL2 complementary DNA and antibiotic selection before injection) were significantly lysed by macrophages of mice treated with LPS, whereas parental or control transfected cells were not (130). The same effect was achieved *in vitro* with the supernatants of CCL2-transfected syngeneic NIH3T3 fibroblasts and LPS, which synergistically activated tumoricidal properties in PEMs against RENCA (renal adenocarcinoma cell line) cells (129). Moreover, alveolar macrophages were isolated after injection of CCL2-transfected fibroblasts or mock-transfected fibroblasts into the tail vein. Cytotoxicity of these macrophages was then tested *in vitro* by coculture with radiolabeled RENCA cells. Cytotoxicity of macrophages against RENCA cells was increased in macrophages gained from mice injected with CCL2-transfected fibroblasts vs. mice injected with control-vector transfected cells. This finding was backed up in a murine *in vivo* model of experimental lung metastases, where subcutaneous administration of CCL2-transfected fibroblasts along with RENCA cells (and subsequent administration of transfected fibroblasts at days 3, 5, and 7 into the original site of injection) reduced tumor size and amount of metastasis to the lung (129). In all the studies of this paragraph, CCL2 (secreted from different cell types, being tumor cells and fibroblasts) is involved in priming macrophages to respond to LPS.

Apart from monocytes and macrophages, also neutrophils have been investigated in the context of influencing tumor progression in the presence of CCL2. A potential antimetastatic effect of CCL2 via activation of neutrophils has been described. *In vitro*, CCL2 was shown to induce killing of cultured MDA-MB231 tumor cells by human neutrophils added as suspension in the presence of granulocyte-CSF (G-CSF). The effect was also shown for naive murine neutrophils on the 4T1 cancer cell line. The cytotoxic effect was underlined by increased H_2_O_2_ production in neutrophils after exogenous CCL2 was added to the media. *In vivo*, knockout of CCL2 in primary tumors of mice injected intradermally with B16-F10 (melanoma) and LLC cells (lung carcinoma) inhibited this neutrophil activation in tumor-bearing mice, and CCL2 knockout tumors showed earlier metastasis *in vivo*. Together, these results indicated that CCL2 released from the primary tumor may have an antimetastatic effect by activation of neutrophils (226). In contrast, Lavender et al. found that naive murine neutrophils were already active on 4T1 cells in the absence of exogenous CCL2 and that CCL2 addition did not increase their cytotoxic potential. Tumor-entrained neutrophils, i.e., neutrophils from tumor-bearing mice, displayed the same behavior. However, the situation was different for the less aggressive 67NR cell line. Naive neutrophils were not active against the 67NR cell line in cocultures, and the addition of exogenous CCL2 did not change that. However, when CCL2 pretreated tumor-naive neutrophils were seeded together with 67NR cells, the neutrophils were then cytotoxic. On the other hand, tumor-entrained neutrophils gained activity against 67NR cells when exogenous CCL2 was added to the coculture. To add to the complexity of the situation, when testing the hypothesis *in vivo*, exogenous CCL2 had an opposite effect on tumors, as it led to increased tumor localization of 67NR cells in mice intravenously injected with 67NR cells and intranasal delivery of CCL2 (227). The findings of the two above studies show that, under certain circumstances, CCL2 is able to confer antitumorigenic and antimetastatic characteristics to neutrophils. However, the exact context of these actions needs to be defined before a definite conclusion can be made.

In summary, direct effect on monocytes, macrophages, and neutrophils (growth inhibitory and tumoricidal) as well as synergistic effects of CCL2 with bacterial endotoxins on activated macrophages were described. These findings characterizing CCL2's effect on reducing tumor cell growth and increased killing via influencing myeloid cells suggest an antitumoricidal role of CCL2. However, these findings are in direct contrast with the publications showing that CCL2 favors tumor progression via M2-macrophage polarization (see *CCL2 Enhances Host Defense, Cellular Cleanup, and Allergic Responses*) and immunosuppression (see *CCL2 Can Confer Immunosuppressive Effects on T Cells via Myeloid Cells*), enhances tumor cell survival and proliferation (8, 74) and metastasis (77) and that high CCL2 correlates with an unfavorable prognosis in several types of cancer such as breast cancer (228, 229), lung adenocarcinoma (230), or pancreatic cancer (231). Moreover, most tumor cell lines produce CCL2 and are able to grow *in vivo* in wild-type as well as CCL2 knockout mice (232, 233). These contrasting effects call for a more detailed investigation in single cancer types, as they might be accountable for the failures in therapeutically targeting CCL2 (see *Discussion and Conclusions*). Potential explanations for the varying effects of CCL2 can be found when studying the molecular aspects of CCL2 biology. This topic is beyond the scope of the review, but the authors would like to point out several potentially important aspects in the following paragraph.

When analyzing the experimental setups (see Table 5 for details), it is of interest to note that the source of CCL2 might have an influence on the functional outcome of CCL2 signaling. PTMs can be dependent on the cellular source and can alter CCL2's function. For example, nitration of its tyrosine residues by reactive nitrogen species, such as peroxynitrite, has been shown to reduce CCL2-mediated monocyte migration in diffusion gradient chemotaxis and transendothelial migration in Transwell assays (234, 235). NO2-CCL2 showed reduced binding to CCR2 and heparan sulfate coreceptors. In addition, NO2-CCL2 was able to antagonize the effects of unmodified CCL2 *in vivo* (235). Furthermore, matrix metalloproteinase (MMP)-2 and MMP-9 have been shown to cleave human CCL2 *in vitro*, which resulted in twofold reduction in THP-1 cell migration (236). Finally, various glycosylated forms of human CCL2 from PBMCs have been characterized, which showed less chemoattractant activity on monocytes (237). Based on these differential effects elicited by PTMs and the multiple effects of CCL2 on myeloid cells described in this review, it seems likely that also CCL2's effects on myeloid cells could be influenced by PTMs. For example, Brault et al. have shown a different outcome when using tumor-derived supernatant containing CCL2 in comparison to recombinant CCL2 (125). The observed effect might be due to different PTMs or to the potential presence of additional stimulatory or inhibitory factors in the supernatant. Moreover, Yoshimura et al. have shown that different cell types serve as the main source of CCL2 in different *in vivo* cancer models (17, 232, 233) and have been comprehensively reviewed in Yoshimura (33).

From a molecular point of view, it is also worth noting the different sizes/molecular weights of human and murine CCL2 (often referred to as JE). Murine CCL2 has a glycosylated 50 amino-acid-long C-terminal tail, which can be cleaved by the protease plasmin generating a chemotactically more potent truncation variant, whereas the N-terminus is more conserved (238, 239). Moreover, a CCL2 homolog, namely, CCL12/MCP-5, exists in mice, which does not exist in humans, which complicates translating finding from *in vivo* mouse models to human diseases (179). Moreover, CCL2 is capable of oligomerizing. Proudfoot et al. suggested that dimerization of CCL2 is required for its function *in vivo* (58) and also Zhang and Rollins concluded that CCL2 functions as dimer (240). In contrast Paolini et al. and Tan et al. state that CCL2 is active as a monomer (241, 242). To add to the complexity, CCL2 can also form heterooligomers with other chemokines (243). On top of that, the active oligomerization state might be different for human and mouse CCL2 as they differ in their C-terminal tail (239) and is influenced by their interaction with glycosaminoglycans (59).

Apart from the molecular aspects of CCL2 biology, the tumor microenvironment is highly complex, and a multitude of molecules and cell types impact on the tumor vs. host-defense battle. CCL2 is certainly not the only culprit, but it is definitely present in the tumor microenvironment and has the potential to impact not only on cancer or stromal cells but also on myeloid cells.

### CCL2 Can Confer Immunosuppressive Effects on T Cells via Myeloid Cells

T cells are also crucial players in host defense in the form of adaptive immune responses, which are tightly interwoven with myeloid-cell-driven innate immunity. Immunosuppressive effects of CCL2 have been described for monocytes/DCs and MDSCs. CCL2 and lipocalin-2, a protein that is induced by CCL2 and is involved in host defense against bacterial infection, cooperatively generated immunoregulatory CD11c+ DCs (DCreg) from CD14+ monocytes *in vitro*. These DCregs displayed suppressive activity as assessed by coculture with autologous CD3+ T cells along with anti-CD3 mAb and measurement of T cell proliferation by colorimetric measurement (WST1 assay). The suppressive effect was accompanied by lowered expression of costimulatory molecules such as HLA-DR and increased expression of immunosuppressive molecules such as PD-L1. Furthermore, the generated DCregs induced FOXP3+ expression on CD4+ T cells, a marker of immunosuppressive Treg phenotype. *In vivo*, CCL2 blockade by either injection of CCL2 small-interfering RNA into tumors of mice injected with Snail transfected B16-F10 murine melanoma cells or treatment with mAb resulted in decreased tumor growth and metastasis (119). Using cocultures of PMN-MDSCs isolated from murine shCCL2 knockdown tumors, and CD4+ T cells, Chun et al. showed that tumor-derived CCL2 confers immunosuppressive effects from PMN-MDSCs on CD4+ T cells. PMN-MDSCs isolated from CCL2 knockdown mouse tumors vs. control tumors showed a decrease in T cell inhibiting ROS production and abolished the decreases in T cell receptor ζ chain expression in CD4+ T cells. The authors found that this effect of CCL2 was mediated by MDSC STAT3 (71). Supportive evidence for the immune-suppressing function of CCL2 via MDSCs was gained in a murine ApcMin/+ *in vivo* model of colon cancer in which anti-CCL2 Ab reduced tumor number compared to isotype control Ab. Adding back exogenous CCL2 by intratumoral injection led to an increase in tumor burden and tumoral MDSC accumulation (71). In addition to affecting T cells, CCL2 secreted from MDSCs seems to have another protumorigenic effect, by modulating angiogenesis. Expression of Rgs2, an inhibitor of cell proliferation and mediator of cell differentiation (244), was shown to be highly upregulated in MDSCs from tumor-bearing mice, and knockout of Rgs2 led to decreased CCL2 expression and reduced tumor growth due to decreased vascularization *in vivo*. *In vitro*, conditioned media from Rgs2^−/−^ MDSCs led to a decrease in angiogenic function of human umbilical vein endothelial cells that could be rescued by exogenous CCL2 (245).

Taken together, these findings show that CCL2 is involved in conveying immunosuppressive properties on T cells via myeloid cells. Therefore, tumors can take advantage of CCL2 secretion by hampering immune defense and increasing angiogenesis at the same time.

## Discussion and Conclusions

CCL2 was one of the first chemokines described and has since been extensively studied for its chemoattractant function (37, 246, 247). Multiple effects of CCL2 are owed to the types of cells it recruits. However, increasing evidence has shown that CCL2 may be far more than merely a guidance cue for leukocytes. In this review, we have summarized how this chemokine influences myeloid cell function and therefore modulates immune responses (Figure 1). Among other effects, CCL2 has been shown to enhance the cell-killing properties of monocytes and macrophages, to enhance survival of macrophages and neutrophils, and to have profound influence on macrophage polarization and corresponding effector molecule secretion. Moreover, immunosuppressive effects of CCL2 on MDSCs have been described, which caused decreased defense against cancers. The summarized data of CCL2's effects show that downstream signaling cascades of CCL2 are not unique to cell migration and often elicit multiple different functions. For example, ERK1, ERK2, and p38 have been shown to be involved in processes as diverse as macrophage activation, tumor cytotoxicity, polarization, and lipid body formation (100, 113, 115). All in all, CCL2 is not a maverick. In a lot of cases, other signaling molecules, such as LPS, or other cell types, such as CD8+ T cells, are involved in mediating the effect of CCL2, and CCL2 acts as the partner in crime. A discussion about CXCL10 and CCL1 as checkpoints was recently published (248), as information about these chemokines in this new role is accumulating. CCL2 was so far only mentioned in the form of a CCL2-MDSC immune checkpoint at the earliest stage of colorectal cancer development in one publication (71). However, the research field about chemokines as checkpoints is growing, and potentially, more findings about CCL2 can be anticipated.

Targeting CCL2 signaling is of high clinical interest due to its involvement in various types of cancer, as well as other difficult to treat diseases, such as atherosclerosis (63), multiple sclerosis (64), and diabetes (65). However, successful clinical modulation of CCL2 and the CCL2/CCR2 axis has remained elusive. Numerous clinical trials (www.clinicaltrials.gov) with anti-CCL2 antibodies, as well as with small molecule CCR2 receptor antagonists, have been conducted. Small molecule CCR2 antagonists were tested for example in metastatic pancreas cancer (PF-04136309, NCT02732938), insulin resistance/type 2 diabetes mellitus (BMS-741672, NCT00699790), and chronic hepatitis C infection (PF-04136309, NCT01226797). Despite the efforts, many small molecule CCR2 antagonist programs have ended in discontinuation for various reasons, such as lack of efficacy or company strategy, whereas some CCR2 antagonists are still in development for instance by Chemocentrix (e.g., CCX872-B, NCT02345408, or CCX140-B NCT03703908). So far, only one small molecule has made it to clinical phase 3, which is the combined small molecule CCR2/CCR5 antagonist Cenicriviroc that is currently tested in a clinical phase 3 study of liver fibrosis in adults with non-alcoholic fatty liver disease (NCT03028740). An antibody against CCL2 (CNTO888, Carlumab), investigated in patients with metastatic castration-resistant prostate cancer and patients with solid tumors, or an antibody against CCR2 (MLN1202, Plozalizumab), investigated in patients with RA, was not successful in clinical trials to date (249, 250). Other approaches that target CCL2 using chemokine mutants (56, 57), a truncated CCL2 analog (251), chemokine fusions (“fusokines”) (252), or spiegelmers (NOX-E36, emapticap pegol) (253) are under investigation. The lack of success in targeting CCL2 has been mirrored with approaches that interfere with the bioactivity of several chemokines in different diseases [reviewed in (254)], which have also so far not lived up to early expectations. A potential pitfall of anti-CCL2/CCR2 treatments could be the functional redundancy of the chemokine ligand and receptor system that allows for compensation of a loss of one chemokine or receptor (59, 255). However, some evidence suggests that the chemokine redundancy that is evident *in vitro* does not occur, at least to the same extent, *in vivo* (256). An alternative explanation suggests that each chemokine/receptor/coreceptor/cell combination has a particular role within a specific physiological/pathological context (257). Other authors argue that inappropriate target selection and ineffective dosing may be more likely mistakes made on the way to CCR targeting therapies (258). Moreover, targeting chemokines and their receptors affects physiological cell migration and development and is therefore a double-edged sword (259). This hurdle could be overcome by making use of novel targeting platforms, such as bispecifics, or applying combination therapies with checkpoint inhibitors (259). At present, our molecular understanding of chemokine presentation, structure–function relationships of chemokines and their (co-)receptors, differential signaling of ligands, and feedback loops regulating the chemokine signaling network is still incomplete and make it currently challenging to interpret or predict the consequences of modulating chemokine behavior *in vivo* (254, 260). This review has shown that it has to be taken into consideration that CCL2 has a significant impact on immune cells that goes beyond attraction. Correspondingly, it has recently been shown that the efficiency of trastuzumab relies on CCL2 levels and monocytes present in the TME in mammary carcinoma *in vivo* (261). A higher CCL2 level also increased the response to chemotherapy (paclitaxel and cisplatin) (262). Therefore, for making a step toward successful targeting of the CCL2/CCR2 axis, it is pivotal not only to understand the effects of CCL2 on cell migration, cancer, and stromal cells but also to consider its effects on immune cells and the underlying molecular mechanisms of action.

Concluding, we have summarized how multifaceted the effects of CCL2 on myeloid cells are. The outcome of the response is highly context, cell type, and time dependent, and in the case of polarization, it can go in different directions. Therefore, the dual role of CCL2 that was pointed out in several contexts, such as tumor progression and immunosurveillance (33, 68, 263, 264), or multifunctionality of chemokine receptors in leukocytes (78), was also confirmed for the effects of the CCL2/CCR2 axis on myeloid cells and calls for a more in depth *in vivo* profiling. Justifiably, in a lot of previous publications about CCL2, the focus has been put on monocyte infiltration, which is the chemokine's best studied function and a prerequisite for impacting on myeloid cells subsequently. With this review, we would like to suggest also taking into account the potential impact of CCL2 on myeloid cell function, as CCL2 might be involved in several subsequent steps of the pathway, including polarization, activation, and survival of myeloid cells.

## Author Contributions

MG, RD, and KM compiled literature information, wrote the review, and edited the manuscript.

### Conflict of Interest

The authors declare that the research was conducted in the absence of any commercial or financial relationships that could be construed as a potential conflict of interest.

## Abbreviations

ACKR: atypical chemokine receptor
BMDM: bone-marrow-derived macrophage
CCR: C–C chemokine receptor
CTL: cytotoxic T lymphocyte
DC: dendritic cell
FBGCs: foreign body giant cells
G-CSF: granulocyte-colony stimulating factor
GM-CSF: granulocyte-macrophage colony-stimulating factor
IFNγ: interferon γ
IL: interleukin
LPS: lipopolysaccharide
mAb: monoclonal antibody
M-CSF: macrophage colony-stimulating factor
MCP-1/CCL2: monocyte chemoattractant protein-1
MDSC: myeloid-derived suppressor cell
oxLDL: oxidized low-density lipoprotein
PBMC: peripheral blood mononuclear cell
PDGF: platelet-derived growth factor
PEM: peritoneal exudate macrophage
RA: rheumatoid arthritis
SAC: *Staphylococcus aureus* Cowan strain 1
SIRS: systemic inflammatory response syndrome
TAM: tumor-associated macrophages
TGFβ: transforming growth factor β
TNFα: tumor necrosis factor α
TPA = PMA: tetradecanoylphorbol acetate
VEGF: vascular endothelial growth factor.

## References

[1] Baggiolini M. Chemokines and leukocyte traffic. Nature. (1998) 392:565–8. 10.1038/33340 9560152

[2] Cushing SD Berliner JA Valente AJ Territo MC Navab M Parhami F. Minimally modified low density lipoprotein induces monocyte chemotactic protein 1 in human endothelial cells and smooth muscle cells. Proc Natl Acad Sci USA. (1990) 87:5134–8. 10.1073/pnas.87.13.5134 1695010 PMC54276

[3] Strieter RM Wiggins R Phan SH Wharram B Showell H Remick DG. Monocyte chemotactic protein gene expression by cytokine-treated human fibroblasts and endothelial cells. Biochem Biophys Res Commun. (1989) 162:694–700. 10.1016/0006-291X(89)92366-8 2787988

[4] Standiford TJ Kunkel SL Phan SH Rollins BJ Strieter RM. Alveolar macrophage-derived cytokines induce monocyte chemoattractant protein-1 expression from human pulmonary type II-like epithelial cells. J Biol Chem. (1991) 266:9912–8. 2033076

[5] Brown Z Strieter RM Neild GH Thompson RC Kunkel SL Westwick J. IL-1 receptor antagonist inhibits monocyte chemotactic peptide 1 generation by human mesangial cells. Kidney Int. (1992) 42:95–101. 10.1038/ki.1992.266 1386129

[6] Barna BP Pettay J Barnett GH Zhou P Iwasaki K Estes ML. Regulation of monocyte chemoattractant protein-1 expression in adult human non-neoplastic astrocytes is sensitive to tumor necrosis factor (TNF) or antibody to the 55-kDa TNF receptor. J Neuroimmunol. (1994) 50:101–7. 10.1016/0165-5728(94)90220-8 8300851

[7] Owen JL Torroella-Kouri M Handel-Fernandez ME Iragavarapu-Charyulu V. GM-CSF up-regulates the expression of CCL2 by T lymphocytes in mammary tumor-bearing mice. Int J Mol Med. (2007) 20:129–36. 10.3892/ijmm.20.1.129 17549399

[8] Loberg RD Day LL Harwood J Ying C John LNS Giles R. CCL2 is a potent regulator of prostate cancer cell migration and proliferation. Neoplasia. (2006) 8:578–86. 10.1593/neo.06280 16867220 PMC1601934

[9] Küper C Beck FX Neuhofer W. Autocrine MCP-1/CCR2 signaling stimulates proliferation and migration of renal carcinoma cells. Oncol Lett. (2016) 12:2201–9. 10.3892/ol.2016.4875 27602164 PMC4998526

[10] Roca H Varsos ZS Sud S Craig MJ Ying C Pienta KJ. CCL2 and interleukin-6 promote survival of human CD11b+ peripheral blood mononuclear cells and induce M2-type macrophage polarization. J Biol Chem. (2009) 284:34342–54. 10.1074/jbc.M109.042671 19833726 PMC2797202

[11] Akhter N Hasan A Shenouda S Wilson A Kochumon S Ali S TLR4/MyD88-mediated CCL2 production by lipopolysaccharide (endotoxin): Implications for metabolic inflammation. J Diab Metabol Disord. 17:77–84. 10.1007/s40200-018-0341-y PMC6154519 30288388

[12] Lee S Lee E Ko E Ham M Lee HM Kim E-S. Tumor-associated macrophages secrete CCL2 and induce the invasive phenotype of human breast epithelial cells through upregulation of ERO1-α and MMP-9. Cancer Lett. (2018) 437:25–34. 10.1016/j.canlet.2018.08.025 30165193

[13] Carson WF Salter-Green SE Scola MM Joshi A Gallagher KA. Enhancement of macrophage inflammatory responses by CCL2 is correlated with increased miR-9 expression and downregulation of the ERK1/2 phosphatase Dusp6. Cell Immunol. (2017) 314:63–72. 10.1016/j.cellimm.2017.02.005 28242024 PMC5425952

[14] Park E-J Kim SA Choi YM Kwon H-K Shim W Lee G. Capric acid inhibits NO production and STAT3 activation during LPS-induced osteoclastogenesis. PLoS ONE. (2011) 6:e27739. 10.1371/journal.pone.0027739 22110749 PMC3218024

[15] Kanayama M Kurotaki D Morimoto J Asano T Matsui Y Nakayama Y. Alpha9 integrin and its ligands constitute critical joint microenvironments for development of autoimmune arthritis. J Immunol. (2009) 182:8015–25. 10.4049/jimmunol.0900725 19494327

[16] Fujimoto H Sangai T Ishii G Ikehara A Nagashima T Miyazaki M. Stromal MCP-1 in mammary tumors induces tumor-associated macrophage infiltration and contributes to tumor progression. Int J Cancer. (2009) 125:1276–84. 10.1002/ijc.24378 19479998

[17] Yoshimura T Imamichi T Weiss JM Sato M Li L Matsukawa A. Induction of monocyte chemoattractant proteins in macrophages via the production of granulocyte/macrophage colony-stimulating factor by breast cancer cells. Front Immunol. (2016) 7:2. 10.3389/fimmu.2016.00002 26834744 PMC4718995

[18] Meda L Bernasconi S Bonaiuto C Sozzani S Zhou D Otvos L. Beta-amyloid (25-35) peptide and IFN-gamma synergistically induce the production of the chemotactic cytokine MCP-1/JE in monocytes and microglial cells. J Immunol. (1996) 157:1213–8. 8757628

[19] Rodriguez-Garcia M Shen Z Barr FD Boesch AW Ackerman ME Kappes JC. Dendritic cells from the human female reproductive tract rapidly capture and respond to HIV. Mucosal Immunol. (2017) 10:531. 10.1038/mi.2016.72 27579858 PMC5332537

[20] Sprokholt JK Kaptein TM van Hamme JL Overmars RJ Gringhuis SI Geijtenbeek TB. RIG-I–like receptor triggering by dengue virus drives dendritic cell immune activation and TH1 differentiation. J Immunol. (2017) 198:4764–71. 10.4049/jimmunol.1602121 28507028

[21] Del Corno M Michienzi A Masotti A Da Sacco L Bottazzo GF Belardelli F CCL2 down-modulation by selected TLR agonist combinations contributes to Th1 polarization in human dendritic cells. Blood. (2009) 114:796–806. 10.1182/blood-2009-01-199406 19465691

[22] Baghestanian M Hofbauer R Kiener HP Bankl HC Wimazal F Willheim M. The c-kit ligand stem cell factor and anti-IgE promote expression of monocyte chemoattractant protein-1 in human lung mast cells. Blood. (1997) 90:4438–49. 10.1182/blood.V90.11.4438 9373254

[23] Venkatesha RT Thangam EB Zaidi AK Ali H. Distinct regulation of C3a-induced MCP-1/CCL2 and RANTES/CCL5 production in human mast cells by extracellular signal regulated kinase and PI3 kinase. Mol Immunol. (2005) 42:581–7. 10.1016/j.molimm.2004.09.009 15607817

[24] Ali H Ahamed J Hernandez-Munain C Baron JL Krangel MS Patel DD. Chemokine production by G protein-coupled receptor activation in a human mast cell line: roles of extracellular signal-regulated kinase and NFAT. J Immunol. (2000) 165:7215–23. 10.4049/jimmunol.165.12.7215 11120854

[25] Yoshimura T Takahashi M. IFN-γ-mediated survival enables human neutrophils to produce MCP-1/CCL2 in response to activation by TLR ligands. J Immunol. (2007) 179:1942–9. 10.4049/jimmunol.179.3.1942 17641061

[26] Hilda JN Das SD. TLR stimulation of human neutrophils lead to increased release of MCP-1, MIP-1α, IL-1β, IL-8 and TNF during tuberculosis. Hum Immunol. (2016) 77:63–7. 10.1016/j.humimm.2015.10.005 26472013

[27] Ahamed J Haribabu B Ali H. Cutting edge: Differential regulation of chemoattractant receptor-induced degranulation and chemokine production by receptor phosphorylation. J Immunol. (2001) 167:3559–63. 10.4049/jimmunol.167.7.3559 11564766

[28] Izumi S Hirai K Miyamasu M Takahashi Y Misaki Y Takaishi T. Expression and regulation of monocyte chemoattractant protein-1 by human eosinophils. Eur J Immunol. (1997) 27:816–24. 10.1002/eji.1830270404 9130630

[29] Van Coillie E Van Damme J Opdenakker G. The MCP/eotaxin subfamily of CC chemokines. Cytokine Growth Factor Rev. (1999) 10:61–86. 10.1016/S1359-6101(99)00005-2 10379912

[30] Kumar SN Boss JM. Site A of the MCP-1 distal regulatory region functions as a transcriptional modulator through the transcription factor NF1. Mol Immunol. (2000) 37:623–32. 10.1016/S0161-5890(00)00097-3 11164890

[31] Luther SA Cyster JG. Chemokines as regulators of T cell differentiation. Nat Immunol. (2001) 2:102. 10.1038/84205 11175801

[32] Choi S You S Kim D Choi SY Kwon HM Kim H-S. Transcription factor NFAT5 promotes macrophage survival in rheumatoid arthritis. J Clin Investig. (2017) 127:954–69. 10.1172/JCI87880 28192374 PMC5330733

[33] Yoshimura T The chemokine MCP-1 (CCL2) in the host interaction with cancer: a foe or ally? Cell Mol Immunol. (2018) 15:335–45. 10.1038/cmi.2017.135 29375123 PMC6052833

[34] Tsaur I Noack A Makarevic J Oppermann E Waaga-Gasser AM Gasser M. CCL2 chemokine as a potential biomarker for prostate cancer: a pilot study. Cancer Res Treat. (2015) 47:306. 10.4143/crt.2014.015 25483747 PMC4398105

[35] Lubowicka E Przylipiak A Zajkowska M Piskór BM Malinowski P Fiedorowicz W. Plasma chemokine CCL2 and its receptor CCR2 concentrations as diagnostic biomarkers for breast cancer patients. BioMed Res Int. (2018) 2018:2124390. 10.1155/2018/2124390 30151375 PMC6091289

[36] Uhlén M Fagerberg L Hallström BM Lindskog C Oksvold P Mardinoglu A. Tissue-based map of the human proteome. Science. (2015) 347:1260419. 10.1126/science.1260419 25613900

[37] Matsushima K Larsen CG DuBois G Oppenheim J. Purification and characterization of a novel monocyte chemotactic and activating factor produced by a human myelomonocytic cell line. J Exp Med. (1989) 169:1485–90. 10.1084/jem.169.4.1485 2926331 PMC2189236

[38] Yoshimura T Robinson EA Tanaka S Appella E Kuratsu JL Leonard EJ. Purification and amino acid analysis of two human glioma-derived monocyte chemoattractants. J Exp Med. (1989) 169:1449–1459. 10.1084/jem.169.4.1449 2926329 PMC2189237

[39] Carr MW Roth SJ Luther E Rose SS Springer TA. Monocyte chemoattractant protein 1 acts as a T-lymphocyte chemoattractant. Proc Natl Acad Sci USA. (1994) 91:3652–6. 10.1073/pnas.91.9.3652 8170963 PMC43639

[40] Frade J Mellado M del Real G Gutierrez-Ramos J Lind P Martinez A. Characterization of the CCR2 chemokine receptor: functional CCR2 receptor expression in B cells. J Immunol. (1997) 159:5576–84. 9548499

[41] Allavena P Bianchi G Zhou D Van Damme J Jílek P Sozzani S. Induction of natural killer cell migration by monocyte chemotactic protein– 1,– 2 and– 3. Eur J Immunol. (1994) 24:3233–6. 10.1002/eji.1830241249 7805752

[42] Conti P Pang X Boucher W Letourneau R Reale M Barbacane RC. Impact of Rantes and MCP-1 chemokines on *in vivo* basophilic cell recruitment in rat skin injection model and their role in modifying the protein and mRNA levels for histidine decarboxylase. Blood. (1997) 89:4120–7. 10.1182/blood.V89.11.4120 9166854

[43] Gendelman HE Ding S Gong N Liu J Ramirez SH Persidsky Y. Monocyte chemotactic protein-1 regulates voltage-gated K+ channels and macrophage transmigration. J Neuroimmune Pharmacol. (2009) 4:47–59. 10.1007/s11481-008-9135-1 19034671 PMC2657224

[44] Zhu K Shen Q Ulrich M Zheng M. Human monocyte-derived dendritic cells expressing both chemotactic cytokines IL-8, MCP-1, RANTES and their receptors, and their selective migration to these chemokines. Chin Med J. (2000) 113:1124–8. 11776150

[45] Huang B Lei Z Zhao J Gong W Liu J Chen Z. CCL2/CCR2 pathway mediates recruitment of myeloid suppressor cells to cancers. Cancer Lett. (2007) 252:86–92. 10.1016/j.canlet.2006.12.012 17257744

[46] Johnston B Burns AR Suematsu M Issekutz TB Woodman RC Kubes P. Chronic inflammation upregulates chemokine receptors and induces neutrophil migration to monocyte chemoattractant protein-1. J Clin Invest. (1999) 103:1269–76. 10.1172/JCI5208 10225970 PMC408354

[47] Charo IF Myers SJ Herman A Franci C Connolly AJ Coughlin SR. Molecular cloning and functional expression of two monocyte chemoattractant protein 1 receptors reveals alternative splicing of the carboxyl-terminal tails. Proc Natl Acad Sci USA. (1994) 91:2752–6. 10.1073/pnas.91.7.2752 8146186 PMC43448

[48] Mellado M Rodriguez-Frade J Aragay A Del Real G Martin A Vila-Coro A. The chemokine monocyte chemotactic protein 1 triggers Janus kinase 2 activation and tyrosine phosphorylation of the CCR2B receptor. J Immunol. (1998) 161:805–13. 9670957

[49] Cambien B Pomeranz M Millet M-A Rossi B Schmid-Alliana A. Signal transduction involved in MCP-1–mediated monocytic transendothelial migration. Blood. (2001) 97:359–66. 10.1182/blood.V97.2.359 11154209

[50] Wain J Kirby J Ali S. Leucocyte chemotaxis: Examination of mitogen-activated protein kinase and phosphoinositide 3-kinase activation by Monocyte Chemoattractant Proteins-1,-2,-3 and-4. Clin Exp Immunol. (2002) 127:436–44. 10.1046/j.1365-2249.2002.01764.x 11966759 PMC1906309

[51] Turner SJ Domin J Waterfield MD Ward SG Westwick J. The CC chemokine monocyte chemotactic peptide-1 activates both the class I p85/p110 phosphatidylinositol 3-kinase and the class II PI3K-C2α. J Biol Chem. (1998) 273:25987–95. 10.1074/jbc.273.40.25987 9748276

[52] Kuang Y Wu Y Jiang H Wu D Selective G protein coupling by CC chemokine receptors. J Biol Chem. (1996) 271:3975–8. 10.1074/jbc.271.8.3975 8626727

[53] Bonecchi R Graham GJ. Atypical chemokine receptors and their roles in the resolution of the inflammatory response. Front Immunol. (2016) 7:224. 10.3389/fimmu.2016.00224 27375622 PMC4901034

[54] Vacchini A Locati M Borroni EM. Overview and potential unifying themes of the atypical chemokine receptor family. J Leukoc Biol. (2016) 99:883–92. 10.1189/jlb.2MR1015-477R 26740381

[55] Nibbs RJ Graham GJ. Immune regulation by atypical chemokine receptors. Nat Rev Immunol. (2013) 13:815. 10.1038/nri3544 24319779

[56] Gerlza T Winkler S Atlic A Zankl C Konya V Kitic N. Designing a mutant CCL2-HSA chimera with high glycosaminoglycan-binding affinity and selectivity. Protein Eng Des Sel. (2015) 28:231–40. 10.1093/protein/gzv025 25969511

[57] Gschwandtner M Piccinini AM Gerlza T Adage T Kungl AJ. Interfering with the CCL2-glycosaminoglycan axis as a potential approach to modulate neuroinflammation. Neurosci Lett. (2016) 626:164–73. 10.1016/j.neulet.2016.05.037 27212623

[58] Proudfoot AEI Handel TM Johnson Z Lau EK LiWang P Clark-Lewis I. Glycosaminoglycan binding and oligomerization are essential for the *in vivo* activity of certain chemokines. Proc Natl Acad Sci USA. (2003) 100:1885–90. 10.1073/pnas.0334864100 12571364 PMC149928

[59] Dyer DP Salanga CL Volkman BF Kawamura T Handel TM. The dependence of chemokine-glycosaminoglycan interactions on chemokine oligomerization. Glycobiology. (2016) 26:312–26. 10.1093/glycob/cwv100 26582609 PMC4736540

[60] Ajuebor MN Flower RJ Hannon R Christie M Bowers K Verity A. Endogenous monocyte chemoattractant protein-1 recruits monocytes in the zymosan peritonitis model. J Leukoc Biol. (1998) 63:108–16. 10.1002/jlb.63.1.108 9469480

[61] Deshmane SL Kremlev S Amini S Sawaya BE. Monocyte chemoattractant protein-1 (MCP-1): an overview. J Interferon Cytokine Res. (2009) 29:313–26. 10.1089/jir.2008.0027 19441883 PMC2755091

[62] Stankovic A Slavic V Stamenkovic B Kamenov B Bojanovic M Mitrovic D. Serum and synovial fluid concentrations of CCL2 (MCP-1) chemokine in patients suffering rheumatoid arthritis and osteoarthritis reflect disease activity. Bratisl. Lek. Listy. (2009) 110:641–6. 20017457

[63] Harrington JR. The role of MCP-1 in atherosclerosis. Stem Cells. (2000) 18:65–6. 10.1634/stemcells.18-1-65 10661575

[64] Mahad DJ Ransohoff RM. The role of MCP-1 (CCL2) and CCR2 in multiple sclerosis and experimental autoimmune encephalomyelitis (EAE). In: Fish E. N. editor. Seminars in immunology. Amsterdam: Elsevier (2003). p. 23–32. 10.1016/S1044-5323(02)00125-2 12495638

[65] Panee J. Monocyte Chemoattractant Protein 1 (MCP-1) in obesity and diabetes. Cytokine. (2012) 60:1–12. 10.1016/j.cyto.2012.06.018 22766373 PMC3437929

[66] Aukrust P Ueland T Müller F Andreassen AK Nordøy I Aas H Elevated circulating levels of CC chemokines in patients with congestive heart failure. Circulation. (1998) 97:1136–43. 10.1161/01.CIR.97.12.1136 9537339

[67] Yadav A Saini V Arora S. MCP-1: chemoattractant with a role beyond immunity: a review. Clin Chim Acta. (2010) 411:1570–9. 10.1016/j.cca.2010.07.006 20633546

[68] O'Connor T Borsig L Heikenwalder M. CCL2-CCR2 signaling in disease pathogenesis. Endocr Metab Immune Disord Drug Targets. (2015) 15:105–18. 10.2174/1871530315666150316120920 25772168

[69] Qian B Li J Zhang H Zhang J Snyder LA Pollard JW CCL2 recruits inflammatory monocytes to facilitate breast tumor metastasis. Cancer Res. (2011) 71(Suppl. 8):2842 10.1158/1538-7445.AM2011-2842 PMC3208506 21654748

[70] Roca H Varsos Z Pienta KJ. CCL2 protects prostate cancer PC3 cells from autophagic death via phosphatidylinositol 3-kinase/AKT-dependent survivin up-regulation. J Biol Chem. (2008) 283:25057–73. 10.1074/jbc.M801073200 18611860 PMC2529129

[71] Chun E Lavoie S Michaud M Gallini CA Kim J Soucy G. CCL2 promotes colorectal carcinogenesis by enhancing polymorphonuclear myeloid-derived suppressor cell population and function. Cell Rep. (2015) 12:244–57. 10.1016/j.celrep.2015.06.024 26146082 PMC4620029

[72] Snyder L Kesavan P Kaiser E Rudnick K McCabe F Millar H Neutralization of CCL2 Inhibits Tumor Angiogenesis and Pancreatic Tumor Growth. Philadelphia, PA: AACR (2007).

[73] Gazzaniga S Bravo AI Guglielmotti A Van Rooijen N Maschi F Vecchi A. Targeting tumor-associated macrophages and inhibition of MCP-1 reduce angiogenesis and tumor growth in a human melanoma xenograft. J Investig Dermatol. (2007) 127:2031–41. 10.1038/sj.jid.5700827 17460736

[74] Fang WB Jokar I Zou A Lambert D Dendukuri P Cheng N. CCL2/CCR2 chemokine signaling coordinates survival and motility of breast cancer cells through Smad3 protein-and p42/44 mitogen-activated protein kinase (MAPK)-dependent mechanisms. J Biol Chem. (2012) 287:36593–608. 10.1074/jbc.M112.365999 22927430 PMC3476325

[75] Eferl R. CCL2 at the crossroad of cancer metastasis. Jak-Stat. (2013) 2:91–105. 10.4161/jkst.23816 24058811 PMC3710324

[76] Borsig L Wolf MJ Roblek M Lorentzen A Heikenwalder M. Inflammatory chemokines and metastasis—tracing the accessory. Oncogene. (2014) 33:3217. 10.1038/onc.2013.272 23851506

[77] Lim SY Yuzhalin AE Gordon-Weeks AN Muschel RJ. Targeting the CCL2-CCR2 signaling axis in cancer metastasis. Oncotarget. (2016) 7:28697–710. 10.18632/oncotarget.7376 26885690 PMC5053756

[78] Lopez-Cotarelo P Gomez-Moreira C Criado-Garcia O Sanchez L Rodriguez-Fernandez JL. Beyond chemoattraction: multifunctionality of chemokine receptors in Leukocytes. Trends Immunol. (2017) 38:927–41. 10.1016/j.it.2017.08.004 28935522

[79] Proudfoot AE Handel TM Graham G. Chemokines–Beyond Chemotaxis. Amsterdam: Elsevier. (2018). 10.1016/j.cyto.2018.04.036 29861383

[80] Daly C Rollins BJ. Monocyte chemoattractant protein-1 (CCL2) in inflammatory disease and adaptive immunity: therapeutic opportunities and controversies. Microcirculation. (2003) 10:247–57. 10.1080/mic.10.3-4.247.257 12851642

[81] Robertson MJ. Role of chemokines in the biology of natural killer cells. J Leukoc Biol. (2002) 71:173–83. 11818437

[82] Jiang Y Beller D Frendl G Graves D. Monocyte chemoattractant protein-1 regulates adhesion molecule expression and cytokine production in human monocytes. J Immunol. (1992) 148:2423–8. 1348518

[83] Braun MC Lahey E Kelsall BL. Selective suppression of IL-12 production by chemoattractants. J Immunol. (2000) 164:3009–17. 10.4049/jimmunol.164.6.3009 10706689

[84] Neumark E Sagi-Assif O Shalmon B Ben-Baruch A Witz IP. Progression of mouse mammary tumors: MCP-1-TNFα cross-regulatory pathway and clonal expression of promalignancy and antimalignancy factors. Int J Cancer. (2003) 106:879–86. 10.1002/ijc.11337 12918065

[85] Flores-Villanueva PO Ruiz-Morales JA Song C-H Flores LM Jo E-K Montaño M. A functional promoter polymorphism in monocyte chemoattractant protein−1 is associated with increased susceptibility to pulmonary tuberculosis. J Exp Med. (2005) 202:1649–58. 10.1084/jem.20050126 16352737 PMC2212957

[86] Vaddi K Newton RC. Regulation of monocyte integrin expression by beta-family chemokines. J Immunol. (1994) 153:4721–32. 7525713

[87] Palframan RT Jung S Cheng G Weninger W Luo Y Dorf M. Inflammatory chemokine transport and presentation in HEV: a remote control mechanism for monocyte recruitment to lymph nodes in inflamed tissues. J Exp Med. (2001) 194:1361–74. 10.1084/jem.194.9.1361 11696600 PMC2195988

[88] Gerszten RE Garcia-Zepeda EA Lim Y-C Yoshida M Ding HA Gimbrone MA Jr. MCP-1 and IL-8 trigger firm adhesion of monocytes to vascular endothelium under flow conditions. Nature. (1999) 398:718. 10.1038/19546 10227295

[89] Maus U Henning S Wenschuh H Mayer K Seeger W Lohmeyer J Role of endothelial MCP-1 in monocyte adhesion to inflamed human endothelium under physiological flow. Am J Physiol Heart Circulat Physiol. (2002) 52:H2584 10.1152/ajpheart.00349.2002 12388329

[90] Locati M Zhou D Luini W Evangelista V Mantovani A Sozzani S. Rapid induction of arachidonic acid release by monocyte chemotactic protein-1 and related chemokines. Role of Ca2+ influx, synergism with platelet-activating factor and significance for chemotaxis. J Biol Chem. (1994) 269:4746–53. 8106442

[91] Rollins BJ Walz A Baggiolini M. Recombinant human MCP-1/JE induces chemotaxis, calcium flux, and the respiratory burst in human monocytes. Blood. (1991) 78:1112–6. 10.1182/blood.V78.4.1112.1112 1868242

[92] Zachariae C Anderson AO Thompson HL Appella E Mantovani A Oppenheim JJ. Properties of monocyte chemotactic and activating factor (MCAF) purified from a human fibrosarcoma cell line. J Exp Med. (1990) 171:2177–82. 10.1084/jem.171.6.2177 2161898 PMC2187957

[93] Rollins BJ Sunday ME. Suppression of tumor formation *in vivo* by expression of the JE gene in malignant cells. Mol Cell Biol. (1991) 11:3125–31. 10.1128/MCB.11.6.3125 2038321 PMC360158

[94] Asano T An T Jia SF Kleinerman ES. Altered monocyte chemotactic and activating factor gene expression in human glioblastoma cell lines increased their susceptibility to cytotoxicity. J Leukoc Biol. (1996) 59:916–24. 10.1002/jlb.59.6.916 8691078

[95] Gu W Yao L Li L Zhang J Place AT Minshall RD. ICAM-1 regulates macrophage polarization by suppressing MCP-1 expression via miR-124 upregulation. Oncotarget. (2017) 8:111882. 10.18632/oncotarget.22948 29340098 PMC5762366

[96] Lumeng CN Bodzin JL Saltiel AR. Obesity induces a phenotypic switch in adipose tissue macrophage polarization. J Clin Invest. (2007) 117:175–84. 10.1172/JCI29881 17200717 PMC1716210

[97] Nio Y Yamauchi T Iwabu M Okada-Iwabu M Funata M Yamaguchi M. Monocyte chemoattractant protein-1 (MCP-1) deficiency enhances alternatively activated M2 macrophages and ameliorates insulin resistance and fatty liver in lipoatrophic diabetic A-ZIP transgenic mice. Diabetologia. (2012) 55:3350–8. 10.1007/s00125-012-2710-2 22983634

[98] Wang Q Ren J Morgan S Liu Z Dou C Liu B. Monocyte chemoattractant protein-1 (MCP-1) regulates macrophage cytotoxicity in abdominal aortic aneurysm. PLoS ONE. (2014) 9:e92053. 10.1371/journal.pone.0092053 24632850 PMC3954911

[99] Mu J RhoA signaling in CCL2-induced macrophage polarization. J Allergy Clin Immunol. (2018) 141:AB114 10.1016/j.jaci.2017.12.363

[100] Sierra-Filardi E Nieto C Dominguez-Soto A Barroso R Sanchez-Mateos P Puig-Kroger A. CCL2 shapes macrophage polarization by GM-CSF and M-CSF: identification of CCL2/CCR2-dependent gene expression profile. J Immunol. (2014) 192:3858–67. 10.4049/jimmunol.1302821 24639350

[101] Roy RM Wüthrich M Klein BS. Chitin elicits CCL2 from airway epithelial cells and induces CCR2-dependent innate allergic inflammation in the lung. J Immunol. (2012) 189:2545–52. 10.4049/jimmunol.1200689 22851704 PMC3424300

[102] Khan UA Hashimi SM Bakr MM Forwood MR Morrison NA. CCL2 and CCR2 are essential for the formation of osteoclasts and foreign body giant cells. J Cell Biochem. (2016) 117:382–9. 10.1002/jcb.25282 26205994

[103] Morrison NA Day CJ Nicholson GC. Dominant negative MCP-1 blocks human osteoclast differentiation. J. Cell. Biochem. (2014) 115:303–12. 10.1002/jcb.24663 23996571

[104] Sul OJ Ke K Kim WK Kim SH Lee SC Kim HJ. Absence of MCP-1 leads to elevated bone mass via impaired actin ring formation. J Cell Physiol. (2012) 227:1619–27. 10.1002/jcp.22879 21678414

[105] Binder NB Niederreiter B Hoffmann O Stange R Pap T Stulnig TM Estrogen-dependent and CC chemokine receptor-2–dependent pathways determine osteoclast behavior in osteoporosis. Nat Med. (2009) 15:417 10.1038/nm.1945 19330010

[106] Shigematsu K Asai A Kobayashi M Herndon DN Suzuki F. Enterococcus faecalis translocation in mice with severe burn injury: a pathogenic role of CCL2 and alternatively activated macrophages (M2aMΦ and M2cMΦ). J Leukoc Biol. (2009) 86:999–1005. 10.1189/jlb.0409235 19622799

[107] Tsuda Y Takahashi H Kobayashi M Hanafusa T Herndon DN Suzuki F. CCL2, a product of mice early after systemic inflammatory response syndrome (SIRS), induces alternatively activated macrophages capable of impairing antibacterial resistance of SIRS mice. J Leukoc Biol. (2004) 76:368–73. 10.1189/jlb.1203645 15123772

[108] Chen M Forrester JV Xu H Dysregulation in retinal para-inflammation and age-related retinal degeneration in CCL2 or CCR2 deficient mice. PLoS ONE. (2011) 6:e22818 10.1371/journal.pone.0022818 21850237 PMC3151263

[109] Chensue SW Warmington KS Ruth JH Sanghi PS Lincoln P Kunkel SL. Role of monocyte chemoattractant protein-1 (MCP-1) in Th1 (mycobacterial) and Th2 (schistosomal) antigen-induced granuloma formation: relationship to local inflammation, Th cell expression, and IL-12 production. J Immunol. (1996) 157:4602–8. 8906839

[110] Tanaka T Terada M Ariyoshi K Morimoto K. Monocyte chemoattractant protein-1/CC chemokine ligand 2 enhances apoptotic cell removal by macrophages through Rac1 activation. Biochem Biophys Res Commun. (2010) 399:677–82. 10.1016/j.bbrc.2010.07.141 20691665

[111] Jürgensen HJ Silva LM Krigslund O van Putten S Madsen DH Behrendt N CCL2/MCP-1 signaling drives extracellular matrix turnover by diverse macrophage subsets. Matrix Biology Plus. (2019) 1:100003 10.1016/j.mbplus.2019.03.002 PMC7852312 33543002

[112] Amano SU Cohen JL Vangala P Tencerova M Nicoloro SM Yawe JC. Local proliferation of macrophages contributes to obesity-associated adipose tissue inflammation. Cell Metab. (2014) 19:162–71. 10.1016/j.cmet.2013.11.017 24374218 PMC3931314

[113] Sodhi A Biswas SK. Monocyte chemoattractant protein-1-induced activation of p42/44 MAPK and c-Jun in murine peritoneal macrophages: a potential pathway for macrophage activation. J Interferon Cytokine Res. (2002) 22:517–26. 10.1089/10799900252981990 12060490

[114] Yang G Meng Y Li W Yong Y Fan Z Ding H. Neuronal MCP-1 mediates microglia recruitment and neurodegeneration induced by the mild impairment of oxidative metabolism. Brain Pathol. (2011) 21:279–97. 10.1111/j.1750-3639.2010.00445.x 21029241 PMC3046243

[115] Pacheco P Vieira-de-Abreu A Gomes RN Barbosa-Lima G Wermelinger LB Maya-Monteiro CM. Monocyte chemoattractant protein-1/CC chemokine ligand 2 controls microtubule-driven biogenesis and leukotriene B4-synthesizing function of macrophage lipid bodies elicited by innate immune response. J Immunol. (2007) 179:8500–8. 10.4049/jimmunol.179.12.8500 18056397

[116] Silva AR Pacheco P Vieira-de-Abreu A Maya-Monteiro CM D'Alegria B Magalhães KG. Lipid bodies in oxidized LDL-induced foam cells are leukotriene-synthesizing organelles: a MCP-1/CCL2 regulated phenomenon. Biochim Biophys Acta Mol Cell Biol Lipids. (2009) 1791:1066–75. 10.1016/j.bbalip.2009.06.004 19573621

[117] Zhao L Shao Q Zhang Y Zhang L He Y Wang L. Human monocytes undergo functional re-programming during differentiation to dendritic cell mediated by human extravillous trophoblasts. Sci Rep. (2016) 6:20409. 10.1038/srep20409 26857012 PMC4746586

[118] Omata N Yasutomi M Yamada A Iwasaki H Mayumi M Ohshima Y. Monocyte chemoattractant protein-1 selectively inhibits the acquisition of CD40 ligand-dependent IL-12-producing capacity of monocyte-derived dendritic cells and modulates Th1 immune response. J Immunol. (2002) 169:4861–6. 10.4049/jimmunol.169.9.4861 12391196

[119] Kudo-Saito C Shirako H Ohike M Tsukamoto N Kawakami Y. CCL2 is critical for immunosuppression to promote cancer metastasis. Clin Exp Metast. (2013) 30:393–405. 10.1007/s10585-012-9545-6 23143679

[120] Fridlender ZG Kapoor V Buchlis G Cheng G Sun J Wang L-CS. Monocyte chemoattractant protein−1 blockade inhibits lung cancer tumor growth by altering macrophage phenotype and activating CD8+ cells. Am J Respir Cell Mol Biol. (2011) 44:230–7. 10.1165/rcmb.2010-0080OC 20395632 PMC3049234

[121] Li N Qin J Lan L Zhang H Liu F Wu Z. PTEN inhibits macrophage polarization from M1 to M2 through CCL2 and VEGF-A reduction and NHERF-1 synergism. Cancer Biol. Ther. (2015) 16:297–306. 10.1080/15384047.2014.1002353 25756512 PMC4622010

[122] Li X Yao W Yuan Y Chen P Li B Li J. Targeting of tumour-infiltrating macrophages via CCL2/CCR2 signalling as a therapeutic strategy against hepatocellular carcinoma. Gut. (2017) 66:157–67. 10.1136/gutjnl-2015-310514 26452628

[123] Yang Z Li H Wang W Zhang J Jia S Wang J. CCL2/CCR2 axis promotes the progression of salivary adenoid cystic carcinoma via recruiting and reprogramming the tumor-associated macrophages. Front Oncol. (2019) 9:231. 10.3389/fonc.2019.00231 31024838 PMC6465613

[124] Kersten K Coffelt SB Hoogstraat M Verstegen NJ Vrijland K Ciampricotti M. Mammary tumor-derived CCL2 enhances pro-metastatic systemic inflammation through upregulation of IL1β in tumor-associated macrophages. Oncoimmunology. (2017) 6:e1334744. 10.1080/2162402X.2017.1334744 28919995 PMC5593698

[125] Brault M Kurt RA. Impact of tumor-derived CCL2 on macrophage effector function. Biomed Res Int. (2005) 2005:37–43. 10.1155/JBB.2005.37 15689637 PMC1138266

[126] Li Y Zheng Y Li T Wang Q Qian J Lu Y. Chemokines CCL2:3, 14 stimulate macrophage bone marrow homing, proliferation, and polarization in multiple myeloma. Oncotarget. (2015) 6:24218–29. 10.18632/oncotarget.4523 26155942 PMC4695181

[127] Huang S Singh RK Xie K Gutman M Berry KK Bucana CD. Expression of theJE/MCP-1 gene suppresses metastatic potential in murine colon carcinoma cells. Cancer Immunol Immunother. (1994) 39:231–8. 10.1007/BF01525986 7954525 PMC11038689

[128] Singh RK Berry K Matsushima K Yasumoto K Fidler IJ. Synergism between human monocyte chemotactic and activating factor and bacterial products for activation of tumoricidal properties in murine macrophages. J Immunol. (1993) 151:2786–93. 8360492

[129] Huang S Xie K Singh RK Gutman M Bar-Eli M. Suppression of tumor growth and metastasis of murine renal adenocarcinoma by syngeneic fibroblasts genetically engineered to secrete the JE/MCP-1 cytokine. J Interferon Cytokine Res. (1995) 15:655–65. 10.1089/jir.1995.15.655 7553238

[130] Nakashima E Kubota Y Matsushita R Ozaki E Ichimura F Kawahara S. Synergistic antitumor interaction of human monocyte chemotactant protein-1 gene transfer and modulator for tumor-infiltrating macrophages. Pharm Res. (1998) 15:685–9. 10.1023/A:1011906600304 9619775

[131] Handel TM Domaille PJ. Heteronuclear (1H, 13C, 15N) NMR assignments and solution structure of the monocyte chemoattractant protein-1 (MCP-1) dimer. Biochemistry. (1996) 35:6569–84. 10.1021/bi9602270 8639605

[132] Jin T Hereld D. Moving toward understanding eukaryotic chemotaxis. Eur J Cell Biol. (2006) 85:905–13. 10.1016/j.ejcb.2006.04.008 16735076

[133] Geissmann F Jung S Littman DR. Blood monocytes consist of two principal subsets with distinct migratory properties. Immunity. (2003) 19:71–82. 10.1016/S1074-7613(03)00174-2 12871640

[134] Gerhardt T Ley K. Monocyte trafficking across the vessel wall. Cardiovasc Res. (2015) 107:321–30. 10.1093/cvr/cvv147 25990461 PMC4592323

[135] Hardy LA Booth TA Lau EK Handel TM Ali S Kirby JA. Examination of MCP-1 (CCL2) partitioning and presentation during transendothelial leukocyte migration. Lab Investig. (2004) 84:81. 10.1038/labinvest.3700007 14647401

[136] Ghousifam N Derakhshan T Gappa-Fahlenkamp H Effects of local concentration gradients of monocyte chemoattractant protein-1 on monocytes adhesion and transendothelial migration in a three-dimensional *in vitro* vascular tissue model. arXiv Preprint. (2019) *arXiv*:1903.05144.

[137] Yamashiro S Takeya M Kuratsu Ji Ushio Y Takahashi K Yoshimura T. Intradermal injection of monocyte chemoattractant protein-1 induces emigration and differentiation of blood monocytes in rat skin. Int Arch Allergy Immunol. (1998) 115:15–23. 10.1159/000023825 9430491

[138] Wood S Jayaraman V Huelsmann EJ Bonish B Burgad D Sivaramakrishnan G. Pro-inflammatory chemokine CCL2 (MCP-1) promotes healing in diabetic wounds by restoring the macrophage response. PLoS ONE. (2014) 9:e91574. 10.1371/journal.pone.0091574 24618995 PMC3950222

[139] Fuentes ME Durham SK Swerdel MR Lewin AC Barton DS Megill JR. Controlled recruitment of monocytes and macrophages to specific organs through transgenic expression of monocyte chemoattractant protein-1. J Immunol. (1995) 155:5769–76. 7499865

[140] Grewal IS Rutledge BJ Fiorillo JA Gu L Gladue RP Flavell RA. Transgenic monocyte chemoattractant protein-1 (MCP-1) in pancreatic islets produces monocyte-rich insulitis without diabetes: abrogation by a second transgene expressing systemic MCP-1. J Immunol. (1997) 159:401–8. 9200479

[141] Gunn MD Nelken NA Liao X Williams LT. Monocyte chemoattractant protein-1 is sufficient for the chemotaxis of monocytes and lymphocytes in transgenic mice but requires an additional stimulus for inflammatory activation. J Immunol. (1997) 158:376–83. 8977213

[142] Lu B Rutledge BJ Gu L Fiorillo J Lukacs NW Kunkel SL. Abnormalities in monocyte recruitment and cytokine expression in monocyte chemoattractant protein 1–deficient mice. J Exp Med. (1998) 187:601–8. 10.1084/jem.187.4.601 9463410 PMC2212142

[143] Takahashi M Galligan C Tessarollo L Yoshimura T Monocyte chemoattractant protein-1 (MCP-1), not MCP-3, is the primary chemokine required for monocyte recruitment in mouse peritonitis induced with thioglycollate or zymosan A. J Immunol. (2009) 183:3463–71. 10.4049/jimmunol.0802812 19641140 PMC7371094

[144] Schittenhelm L Hilkens CM Morrison VL. β2 integrins as regulators of dendritic cell, monocyte, and macrophage function. Front Immunol. (2017) 8:1866. 10.3389/fimmu.2017.01866 29326724 PMC5742326

[145] Mazzone A Ricevuti G. Leukocyte CD11/CD18 integrins: biological and clinical relevance. Haematologica. (1995) 80:161–75. 7628754

[146] Yi L Chandrasekaran P Venkatesan S. TLR signaling paralyzes monocyte chemotaxis through synergized effects of p38 MAPK and global Rap-1 activation. PLoS ONE. (2012) 7:e30404. 10.1371/journal.pone.0030404 22347375 PMC3276499

[147] Grenon SM Aguado-Zuniga J Hatton JP Owens CD Conte MS Hughes-Fulford M. Effects of fatty acids on endothelial cells: inflammation and monocyte adhesion. J Surg Res. (2012) 177:e35–43. 10.1016/j.jss.2011.11.874 22572621 PMC3756552

[148] Lefkowith JB Lennartz MR Rogers M Morrison A Brown EJ. Phospholipase activation during monocyte adherence and spreading. J Immunol. (1992) 149:1729–35. 1506690

[149] Varol C Mildner A Jung S. Macrophages: development and tissue specialization. Annu Rev Immunol. (2015) 33:643–75. 10.1146/annurev-immunol-032414-112220 25861979

[150] Sica A Mantovani A. Macrophage plasticity and polarization: *in vivo* veritas. J Clin Invest. (2012) 122:787–95. 10.1172/JCI59643 22378047 PMC3287223

[151] Mantovani A Sica A Sozzani S Allavena P Vecchi A Locati M. The chemokine system in diverse forms of macrophage activation and polarization. Trends Immunol. (2004) 25:677–86. 10.1016/j.it.2004.09.015 15530839

[152] Ruytinx P Proost P Van Damme J Struyf S. Chemokine-induced macrophage polarization in inflammatory conditions. Front Immunol. (2018) 9:1930. 10.3389/fimmu.2018.01930 30245686 PMC6137099

[153] Martinez FO Sica A Mantovani A Locati M. Macrophage activation and polarization. Front Biosci. (2008) 13:453–61. 10.2741/2692 17981560

[154] Raes G Van den Bergh R De Baetselier P Ghassabeh GH Arginase-1 and Ym1 are markers for murine, but not human, alternatively activated myeloid cells. J Immunol. (2005) 174:6561–2. 10.4049/jimmunol.174.11.6561 15905489

[155] Murray PJ. Macrophage polarization. Annu Rev Physiol. (2017) 79:541–66. 10.1146/annurev-physiol-022516-034339 27813830

[156] Quintin J Cheng S-C van der Meer JW Netea MG. Innate immune memory: towards a better understanding of host defense mechanisms. Curr Opin Immunol. (2014) 29:1–7. 10.1016/j.coi.2014.02.006 24637148

[157] Alvarez-Errico D Vento-Tormo R Sieweke M Ballestar E. Epigenetic control of myeloid cell differentiation, identity and function. Nat Rev Immunol. (2015) 15:7. 10.1038/nri3777 25534619

[158] Banchereau J Briere F Caux C Davoust J Lebecque S Liu Y-J. Immunobiology of dendritic cells. Annu Rev Immunol. (2000) 18:767–811. 10.1146/annurev.immunol.18.1.767 10837075

[159] Roca L Di Paolo S Petruzzelli V Grandaliano G Ranieri E Schena FP. Dexamethasone modulates interleukin-12 production by inducing monocyte chemoattractant protein-1 in human dendritic cells. Immunol Cell Biol. (2007) 85:610–6. 10.1038/sj.icb.7100108 17700511

[160] Gabrilovich DI. Myeloid-derived suppressor cells. Cancer Immunol Res. (2017) 5:3–8. 10.1158/2326-6066.CIR-16-0297 28052991 PMC5426480

[161] Zhang Y Qu D Sun J Zhao L Wang Q Shao Q. Human trophoblast cells induced MDSCs from peripheral blood CD14+ myelomonocytic cells via elevated levels of CCL2. Cell Mol Immunol. (2016) 13:615. 10.1038/cmi.2015.41 26027727 PMC5037277

[162] Fujisaka Y Iwata T Tamai K Nakamura M Mochizuki M Shibuya R. Long non-coding RNA HOTAIR up-regulates chemokine (C-C motif) ligand 2 and promotes proliferation of macrophages and myeloid-derived suppressor cells in hepatocellular carcinoma cell lines. Oncol Lett. (2018) 15:509–14. 10.3892/ol.2017.7322 29387231 PMC5768083

[163] Yavropoulou M Yovos J. Osteoclastogenesis–current knowledge and future perspectives. J Musculoskelet Neuronal Interact. (2008) 8:204–16. 18799853

[164] Kim MS Day CJ Morrison NA MCP-1 is induced by receptor activator of nuclear factor-κB ligand, promotes human osteoclast fusion, and rescues granulocyte macrophage colony-stimulating factor suppression of osteoclast formation. J Biol Chem. (2005) 280:16163–9. 10.1074/jbc.M412713200 15722361

[165] Miyamoto K Ninomiya K Sonoda K-H Miyauchi Y Hoshi H Iwasaki R. MCP-1 expressed by osteoclasts stimulates osteoclastogenesis in an autocrine/paracrine manner. Biochem Biophys Res Commun. (2009) 383:373–7. 10.1016/j.bbrc.2009.04.020 19364494

[166] Siddiqui JA Partridge NC. CCL2/monocyte chemoattractant protein 1 and parathyroid hormone action on bone. Front Endocrinol. (2017) 8:49. 10.3389/fendo.2017.00049 28424660 PMC5372820

[167] Kyriakides TR Foster MJ Keeney GE Tsai A Giachelli CM Clark-Lewis I. The CC chemokine ligand, CCL2/MCP1, participates in macrophage fusion and foreign body giant cell formation. Am J Pathol. (2004) 165:2157–66. 10.1016/S0002-9440(10)63265-8 15579457 PMC1618731

[168] Denholm EM Stankus GP. Changes in the expression of MCP-1 receptors on monocytic THP-1 cells following differentiation to macrophages with phorbol myristate acetate. Cytokine. (1995) 7:436–40. 10.1006/cyto.1995.0059 7578981

[169] Fantuzzi L Borghi P Ciolli V Pavlakis G Belardelli F Gessani S. Loss of CCR2 expression and functional response to monocyte chemotactic protein (MCP-1) during the differentiation of human monocytes: role of secreted MCP-1 in the regulation of the chemotactic response. Blood. (1999) 94:875–83. 10.1182/blood.V94.3.875.415k28_875_883 10419877

[170] Yoshimura T. cDNA cloning of guinea pig monocyte chemoattractant protein-1 and expression of the recombinant protein. J Immunol. (1993) 150:5025–32. 8496603

[171] Motley MP Madsen DH Jürgensen HJ Spencer DE Szabo R Holmbeck K. A CCR2 macrophage endocytic pathway mediates extravascular fibrin clearance *in vivo*. Blood. (2016) 127:1085–96. 10.1182/blood-2015-05-644260 26647393 PMC4778161

[172] Liu J Xue Y Dong D Xiao C Lin C Wang H. CCR2– and CCR2+ corneal macrophages exhibit distinct characteristics and balance inflammatory responses after epithelial abrasion. Mucosal Immunol. (2017) 10:1145–59. 10.1038/mi.2016.139 28120849 PMC5562841

[173] Bajpai G Bredemeyer A Li W Zaitsev K Koenig AL Lokshina I. Tissue resident CCR2– and CCR2+ cardiac macrophages differentially orchestrate monocyte recruitment and fate specification following myocardial injury. Circ Res. (2019) 124:263–78. 10.1161/CIRCRESAHA.118.314028 30582448 PMC6626616

[174] Johnson Z Proudfoot AE Handel TM. Interaction of chemokines and glycosaminoglycans: a new twist in the regulation of chemokine function with opportunities for therapeutic intervention. Cytokine Growth Factor Rev. (2005) 16:625–36. 10.1016/j.cytogfr.2005.04.006 15990353

[175] Handel TM Johnson Z Rodrigues DH dos Santos AC Cirillo R Muzio V. An engineered monomer of CCL2 has anti-inflammatory properties emphasizing the importance of oligomerization for chemokine activity *in vivo*. J Leukoc Biol. (2008) 84:1101–8. 10.1189/jlb.0108061 18662971 PMC2538597

[176] Piccinini AM Knebl K Rek A Wildner G Diedrichs-Möhring M Kungl AJ. Rationally evolving MCP-1/CCL2 into a decoy protein with potent anti-inflammatory activity *in vivo*. J Biol Chem. (2010) 285:8782–92. 10.1074/jbc.M109.043299 20097750 PMC2838300

[177] Said N Sanchez-Carbayo M Smith SC Theodorescu D. RhoGDI2 suppresses lung metastasis in mice by reducing tumor versican expression and macrophage infiltration. J Clin Invest. (2012) 122:1503–18. 10.1172/JCI61392 22406535 PMC3314474

[178] Masuda A Yasuoka H Satoh T Okazaki Y Yamaguchi Y Kuwana M. Versican is upregulated in circulating monocytes in patients with systemic sclerosis and amplifies a CCL2-mediated pathogenic loop. Arthritis Res Ther. (2013) 15:R74. 10.1186/ar4251 23845159 PMC3979134

[179] Sarafi MN Garcia-Zepeda EA MacLean JA Charo IF Luster AD. Murine monocyte chemoattractant protein (MCP)-5: a novel CC chemokine that is a structural and functional homologue of human MCP-1. J Exp Med. (1997) 185:99–110. 10.1084/jem.185.1.99 8996246 PMC2196097

[180] Bachelerie F Ben-Baruch A Burkhardt AM Combadiere C Farber JM Graham GJ. International union of basic and clinical pharmacology. [corrected] LXXXIX Update on the extended family of chemokine receptors and introducing a new nomenclature for atypical chemokine receptors. Pharmacol Rev. (2014) 66:1–79. 10.1124/pr.113.007724 24218476 PMC3880466

[181] Pei X Sun Q Zhang Y Wang P Peng X Guo C. PC3-secreted microprotein is a novel chemoattractant protein and functions as a high-affinity ligand for CC chemokine receptor 2. J Immunol. (2014) 192:1878–86. 10.4049/jimmunol.1300758 24442440

[182] Chen X Wang Y Nelson D Tian S Mulvey E Patel B. CCL2/CCR2 regulates the tumor microenvironment in HER-2/neu-driven mammary carcinomas in mice. PLoS ONE. (2016) 11:e0165595. 10.1371/journal.pone.0165595 27820834 PMC5098736

[183] Al-Aoukaty A Schall T Maghazachi A. Differential coupling of CC chemokine receptors to multiple heterotrimeric G proteins in human interleukin-2-activated natural killer cells. Blood. (1996) 87:4255–60. 10.1182/blood.V87.10.4255.bloodjournal87104255 8639784

[184] Arai H Charo IF. Differential regulation of G-protein-mediated signaling by chemokine receptors. J Biol Chem. (1996) 271:21814–9. 10.1074/jbc.271.36.21814 8702980

[185] Zhou L Azfer A Niu J Graham S Choudhury M Adamski FM. Monocyte chemoattractant protein-1 induces a novel transcription factor that causes cardiac myocyte apoptosis and ventricular dysfunction. Circ Res. (2006) 98:1177–85. 10.1161/01.RES.0000220106.64661.71 16574901 PMC1523425

[186] Kapoor N Niu J Saad Y Kumar S Sirakova T Becerra E. Transcription factors STAT6 and KLF4 implement macrophage polarization via the dual catalytic powers of MCPIP. J Immunol. (2015) 194:6011–23. 10.4049/jimmunol.1402797 25934862 PMC4458412

[187] Matsushita K Takeuchi O Standley DM Kumagai Y Kawagoe T Miyake T. Zc3h12a is an RNase essential for controlling immune responses by regulating mRNA decay. Nature. (2009) 458:1185–90. 10.1038/nature07924 19322177

[188] Takeuchi O. Endonuclease Regnase-1/Monocyte chemotactic protein-1-induced protein-1 (MCPIP1) in controlling immune responses and beyond. Wiley Interdiscipl Rev RNA. (2018) 9:e1449. 10.1002/wrna.1449 28929622

[189] Zheng Y Cai Z Wang S Zhang X Qian J Hong S. Macrophages are an abundant component of myeloma microenvironment and protect myeloma cells from chemotherapy drug–induced apoptosis. Blood. (2009) 114:3625–8. 10.1182/blood-2009-05-220285 19710503 PMC2766678

[190] Gieni RS Li Y HayGlass KT Comparison of [3H] thymidine incorporation with MTT-and MTS-based bioassays for human and murine IL-2 and IL-4 analysis tetrazolium assays provide markedly enhanced sensitivity. J Immunol Methods. (1995) 187:85–93. 10.1016/0022-1759(95)00170-F 7490461

[191] Jenkins SJ Ruckerl D Cook PC Jones LH Finkelman FD van Rooijen N. Local macrophage proliferation, rather than recruitment from the blood, is a signature of TH2 inflammation. Science. (2011) 332:1284–8. 10.1126/science.1204351 21566158 PMC3128495

[192] Sieweke MH Allen JE. Beyond stem cells: self-renewal of differentiated macrophages. Science. (2013) 342:1242974. 10.1126/science.1242974 24264994

[193] Teng TS Ji AL Ji XY Li YZ. Neutrophils and immunity: from bactericidal action to being conquered. J Immunol Res. (2017) 2017:9671604. 10.1155/2017/9671604 28299345 PMC5337389

[194] Yang EJ Choi E Ko J Kim DH Lee JS Kim IS. Differential effect of CCL2 on constitutive neutrophil apoptosis between normal and asthmatic subjects. J Cell Physiol. (2012) 227:2567–77. 10.1002/jcp.22995 21898402

[195] Sakamoto C Suzuki K Hato F Akahori M Hasegawa T Hino M. Antiapoptotic effect of granulocyte colony-stimulating factor, granulocyte-macrophage colony-stimulating factor, and cyclic AMP on human neutrophils: protein synthesis-dependent and protein synthesis-independent mechanisms and the role of the Janus kinase-STAT pathway. Int J Hematol. (2003) 77:60–70. 10.1007/BF02982604 12568301

[196] Burn T Petrovick M Hohaus S Rollins B Tenen D. Monocyte chemoattractant protein-1 gene is expressed in activated neutrophils and retinoic acid-induced human myeloid cell lines. Blood. (1994) 84:2776–83. 10.1182/blood.V84.8.2776.2776 7919389

[197] Smigiel KS Parks WC. Macrophages, wound healing, and fibrosis: recent insights. Curr Rheumatol Rep. (2018) 20:17. 10.1007/s11926-018-0725-5 29550962

[198] Ravanan P Srikumar IF Talwar P. Autophagy: the spotlight for cellular stress responses. Life Sci. (2017) 188:53–67. 10.1016/j.lfs.2017.08.029 28866100

[199] Kabeya Y Mizushima N Ueno T Yamamoto A Kirisako T Noda T. LC3, a mammalian homologue of yeast Apg8p, is localized in autophagosome membranes after processing. EMBO J. (2000) 19:5720–8. 10.1093/emboj/19.21.5720 11060023 PMC305793

[200] Thomas DC. The phagocyte respiratory burst: historical perspectives and recent advances. Immunol Lett. (2017) 192:88–96. 10.1016/j.imlet.2017.08.016 28864335

[201] Krystel-Whittemore M Dileepan KN Wood JG. Mast cell: a multi-functional master cell. Front Immunol. (2016) 6:620. 10.3389/fimmu.2015.00620 26779180 PMC4701915

[202] Alam R Kumar D Anderson-Walters D Forsythe PA. Macrophage inflammatory protein-1 alpha and monocyte chemoattractant peptide-1 elicit immediate and late cutaneous reactions and activate murine mast cells *in vivo*. J Immunol. (1994) 152:1298–303. 8301133

[203] Campbell EM Charo IF Kunkel SL Strieter RM Boring L Gosling J Monocyte chemoattractant protein-1 mediates cockroach allergen-induced bronchial hyperreactivity in normal but not CCR2–/– mice: the role of mast cells. J Immunol. (1999) 163:2160–7. 10438957

[204] Tominaga T Miyazaki D Sasaki SI Mihara S Komatsu N Yakura K. Blocking mast cell–mediated type I hypersensitivity in experimental allergic conjunctivitis by monocyte chemoattractant protein-1/CCR2. Invest Ophthalmol Vis Sci. (2009) 50:5181–8. 10.1167/iovs.09-3637 19553621

[205] Conti P Boucher W Letourneau R Feliciani C Reale M Barbacane R Monocyte chemotactic protein-1 provokes mast cell aggregation and [3H] 5HT release. Immunology. (1995) 86:434. 8550082 PMC1383948

[206] Lv J Huang Y Zhu S Yang G Zhang Y Leng J. MCP-1-induced histamine release from mast cells is associated with development of interstitial cystitis/bladder pain syndrome in rat models. Mediators Inflamm. (2012) 2012:358184. 10.1155/2012/358184 23049171 PMC3459284

[207] Sriaroon P Ballow M. Biological modulators in eosinophilic diseases. Clin Rev Allergy Immunol. (2016) 50:252–72. 10.1007/s12016-014-8444-9 25129490

[208] Schwartz C Eberle JU Voehringer D. Basophils in inflammation. Eur J Pharmacol. (2016) 778:90–5. 10.1016/j.ejphar.2015.04.049 25959388

[209] Alam R Lett-Brown M Forsythe P Anderson-Walters D Kenamore C Kormos C. Monocyte chemotactic and activating factor is a potent histamine-releasing factor for basophils. J Clin Invest. (1992) 89:723–8. 10.1172/JCI115648 1371775 PMC442914

[210] Kuna P Reddigari S Rucinski D Oppenheim J Kaplan A. Monocyte chemotactic and activating factor is a potent histamine-releasing factor for human basophils. J Exp Med. (1992) 175:489–93. 10.1084/jem.175.2.489 1370686 PMC2119123

[211] Bischoff SC Krieger M Brunner T Rot A Tscharner VV Baggiolini M. RANTES and related chemokines activate human basophil granulocytes through different G protein-coupled receptors. Eur J Immunol. (1993) 23:761–7. 10.1002/eji.1830230329 7680615

[212] Bischoff SC Krieger M Brunner T Dahinden CA. Monocyte chemotactic protein 1 is a potent activator of human basophils. J Exp Med. (1992) 175:1271–5. 10.1084/jem.175.5.1271 1569397 PMC2119199

[213] Dvorak AM Schroeder JT MacGlashan DW Jr Bryan KP Morgan ES Lichtenstein LM Comparative ultrastructural morphology of human basophils stimulated to release histamine by anti-IgE, recombinant IgE-dependent histamine-releasing factor, or monocyte chemotactic protein-1. J Allergy Clin Immunol. (1996) 98:355–70. 10.1016/S0091-6749(96)70160-4 8757213

[214] Weber M Uguccioni M Baggiolini M Clark-Lewis I Dahinden CA. Deletion of the NH2-terminal residue converts monocyte chemotactic protein 1 from an activator of basophil mediator release to an eosinophil chemoattractant. J Exp Med. (1996) 183:681–5. 10.1084/jem.183.2.681 8627182 PMC2192454

[215] Oliveira SH Lira S Martinez AC Wiekowski M Sullivan L Lukacs NW. Increased responsiveness of murine eosinophils to MIP-1β (CCL4) and TCA-3 (CCL1) is mediated by their specific receptors, CCR5 and CCR8. J Leukoc Biol. (2002) 71:1019–25. 12050188

[216] Komatsu N Miyazaki D Tominaga T Kuo C-H Namba S Takeda S. Transcriptional analyses before and after suppression of immediate hypersensitivity reactions by CCR3 blockade in eyes with experimental allergic conjunctivitis. Invest Ophthalmol Vis Sci. (2008) 49:5307–13. 10.1167/iovs.08-2154 18658092

[217] Henriques MDGMD Penido C γ*δ* T lymphocytes coordinate eosinophil influx during allergic responses. Front Pharmacol. (2012) 3:200 10.3389/fphar.2012.00200 23316161 PMC3540995

[218] Welte MA Gould AP. Lipid droplet functions beyond energy storage. Biochim Biophys Acta Mol Cell Biol Lipids. (2017) 1862:1260–72. 10.1016/j.bbalip.2017.07.006 28735096 PMC5595650

[219] Weagel E Smith C Liu P Robison R O'Neill K Macrophage polarization and its role in cancer. J Clin Cell Immunol. (2015) 6:338 10.4172/2155-9899.1000338

[220] Farhat K Ulmer AJ Jungi TW. A potential test system for detecting contaminations by bacterial lipoproteins. Vet Immunol Immunopathol. (2012) 145:66–73. 10.1016/j.vetimm.2011.10.009 22133281

[221] Schwarz H Schmittner M Duschl A Horejs-Hoeck J. Residual endotoxin contaminations in recombinant proteins are sufficient to activate human CD1c+ dendritic cells. PLoS ONE. (2014) 9:e113840. 10.1371/journal.pone.0113840 25478795 PMC4257590

[222] Coffelt SB Kersten K Doornebal CW Weiden J Vrijland K Hau C-S. IL-17-producing γ*δ* T cells and neutrophils conspire to promote breast cancer metastasis. Nature. (2015) 522:345. 10.1038/nature14282 25822788 PMC4475637

[223] Zhao Y Niu C Cui J Gamma-delta (γδ) T cells: friend or foe in cancer development? J Transl Med. (2018) 16:3 10.1186/s12967-018-1491-x 29316940 PMC5761189

[224] Amatya N Garg AV Gaffen SL. IL-17 signaling: the yin and the yang. Trends Immunol. (2017) 38:310–22. 10.1016/j.it.2017.01.006 28254169 PMC5411326

[225] Alon R Shulman Z. Chemokine triggered integrin activation and actin remodeling events guiding lymphocyte migration across vascular barriers. Exp Cell Res. (2011) 317:632–41. 10.1016/j.yexcr.2010.12.007 21376176

[226] Granot Z Henke E Comen EA King TA Norton L Benezra R. Tumor entrained neutrophils inhibit seeding in the premetastatic lung. Cancer Cell. (2011) 20:300–14. 10.1016/j.ccr.2011.08.012 21907922 PMC3172582

[227] Lavender N Yang J Chen S-C Sai J Johnson CA Owens P. The Yin/Yan of CCL2: a minor role in neutrophil anti-tumor activity *in vitro* but a major role on the outgrowth of metastatic breast cancer lesions in the lung *in vivo*. BMC Cancer. (2017) 17:88. 10.1186/s12885-017-3074-2 28143493 PMC5286656

[228] Ueno T Toi M Saji H Muta M Bando H Kuroi K. Significance of macrophage chemoattractant protein-1 in macrophage recruitment, angiogenesis, and survival in human breast cancer. Clin Cancer Res. (2000) 6:3282–9. 10955814

[229] Wang J Zhuang Z-G Xu S-F He Q Shao Y-G Ji M. Expression of CCL2 is significantly different in five breast cancer genotypes and predicts patient outcome. Int J Clin Exp Med. (2015) 8:15684. 26629063 PMC4658952

[230] Li L Liu YD Zhan YT Zhu YH Li Y Xie D. High levels of CCL2 or CCL4 in the tumor microenvironment predict unfavorable survival in lung adenocarcinoma. Thoracic Cancer. (2018) 9:775–84. 10.1111/1759-7714.12643 29722145 PMC6026602

[231] Sanford DE Belt BA Panni RZ Mayer A Deshpande AD Carpenter D. Inflammatory monocyte mobilization decreases patient survival in pancreatic cancer: a role for targeting the CCL2/CCR2 axis. Clin Cancer Res. (2013) 19:3404–15. 10.1158/1078-0432.CCR-13-0525 23653148 PMC3700620

[232] Yoshimura T Howard OZ Ito T Kuwabara M Matsukawa A Chen K. Monocyte chemoattractant protein-1/CCL2 produced by stromal cells promotes lung metastasis of 4T1 murine breast cancer cells. PLoS ONE. (2013) 8:e58791. 10.1371/journal.pone.0058791 23527025 PMC3601078

[233] Yoshimura T Liu M Chen X Li L Wang JM. Crosstalk between tumor cells and macrophages in stroma renders tumor cells as the primary source of MCP-1/CCL2 in Lewis lung carcinoma. Front Immunol. (2015) 6:332. 10.3389/fimmu.2015.00332 26167165 PMC4481164

[234] Sato E Simpson KL Grisham MB Koyama S Robbins RA. Effects of reactive oxygen and nitrogen metabolites on MCP-1-induced monocyte chemotactic activity *in vitro*. Am J Physiol Lung Cell Mol Physiol. (1999) 277:L543–9. 10.1152/ajplung.1999.277.3.L543 10484461

[235] Barker CE Thompson S O'boyle G Lortat-Jacob H Sheerin NS Ali S. CCL2 nitration is a negative regulator of chemokine-mediated inflammation. Sci Rep. (2017) 7:44384. 10.1038/srep44384 28290520 PMC5349559

[236] Denney H Clench MR Woodroofe MN. Cleavage of chemokines CCL2 and CXCL10 by matrix metalloproteinases-2 and-9: implications for chemotaxis. Biochem Biophys Res Commun. (2009) 382:341–7. 10.1016/j.bbrc.2009.02.164 19281798

[237] Proost P Struyf S Couvreur M Lenaerts J-P Conings R Menten P. Posttranslational modifications affect the activity of the human monocyte chemotactic proteins MCP-1 and MCP-2: identification of MCP-2 (6–76) as a natural chemokine inhibitor. J Immunol. (1998) 160:4034–41. 9558113

[238] Yao Y Tsirka SE. The C terminus of mouse monocyte chemoattractant protein 1 (MCP1) mediates MCP1 dimerization while blocking its chemotactic potency. J Biol Chem. (2010) 285:31509–16. 10.1074/jbc.M110.124891 20682771 PMC2951225

[239] Yao Y Tsirka SE. Mouse monocyte chemoattractant protein 1 (MCP1) functions as a monomer. Int J Biochem Cell Biol. (2014) 55:51–9. 10.1016/j.biocel.2014.08.002 25130440 PMC4252872

[240] Zhang Y Rollins BJ. A dominant negative inhibitor indicates that monocyte chemoattractant protein 1 functions as a dimer. Mol Cell Biol. (1995) 15:4851–5. 10.1128/MCB.15.9.4851 7651403 PMC230730

[241] Paolini JF Willard D Consler T Luther M Krangel MS. The chemokines IL-8, monocyte chemoattractant protein-1, and I-309 are monomers at physiologically relevant concentrations. J Immunol. (1994) 153:2704–17. 8077676

[242] Tan JH Canals M Ludeman JP Wedderburn J Boston C Butler SJ. Design and receptor interactions of obligate dimeric mutant of chemokine monocyte chemoattractant protein-1 (MCP-1). J Biol Chem. (2012) 287:14692–702. 10.1074/jbc.M111.334201 22396538 PMC3340267

[243] Crown SE Yu Y Sweeney MD Leary JA Handel TM. Heterodimerization of CCR2 chemokines and regulation by glycosaminoglycan binding. J Biol Chem. (2006) 281:25438–46. 10.1074/jbc.M601518200 16803905

[244] Schwäble J Choudhary C Thiede C Tickenbrock L Sargin B Steur C. RGS2 is an important target gene of Flt3-ITD mutations in AML and functions in myeloid differentiation and leukemic transformation. Blood. (2005) 105:2107–14. 10.1182/blood-2004-03-0940 15536149

[245] Boelte KC Gordy LE Joyce S Thompson MA Yang L Lin PC. Rgs2 mediates pro-angiogenic function of myeloid derived suppressor cells in the tumor microenvironment via upregulation of MCP-1. PLoS ONE. (2011) 6:e18534. 10.1371/journal.pone.0018534 21494556 PMC3073977

[246] Altman LC Snyderman R Oppenheim JJ Mergenhagen SE. A human mononuclear leukocyte chemotactic factor: characterization, specificity and kinetics of production by homologous leukocytes. J Immunol. (1973) 110:801–10. 4688920

[247] Yoshimura T Robinson E Tanaka S Appella E Leonard E. Purification and amino acid analysis of two human monocyte chemoattractants produced by phytohemagglutinin-stimulated human blood mononuclear leukocytes. J Immunol. (1989) 142:1956–62. 2921521

[248] Karin N. Chemokines and cancer: new immune checkpoints for cancer therapy. Curr Opin Immunol. (2018) 51:140–5. 10.1016/j.coi.2018.03.004 29579623

[249] Vergunst CE Gerlag DM Lopatinskaya L Klareskog L Smith M Van den Bosch F. Modulation of CCR2 in rheumatoid arthritis: a double-blind, randomized, placebo-controlled clinical trial. Arthrit Rheumat. (2008) 58:1931–9. 10.1002/art.23591 18576354

[250] Pienta KJ Machiels J-P Schrijvers D Alekseev B Shkolnik M Crabb SJ. Phase 2 study of carlumab (CNTO 888), a human monoclonal antibody against CC-chemokine ligand 2 (CCL2), in metastatic castration-resistant prostate cancer. Invest New Drugs. (2013) 31:760–8. 10.1007/s10637-012-9869-8 22907596

[251] Kalderén C Forsgren M Karlström U Stefansson K Svensson R Berglund MM. A truncated analogue of CCL2 mediates anti-fibrotic effects on murine fibroblasts independently of CCR2. Biochem Pharmacol. (2012) 83:644–52. 10.1016/j.bcp.2011.12.001 22177985

[252] Rafei M Berchiche YA Birman E Boivin M-N Young YK Wu JH. An engineered GM-CSF-CCL2 fusokine is a potent inhibitor of CCR2-driven inflammation as demonstrated in a murine model of inflammatory arthritis. J Immunol. (2009) 183:1759–66. 10.4049/jimmunol.0900523 19592643

[253] Menne J Eulberg D Beyer D Baumann M Saudek F Valkusz Z CC motif-ligand 2 inhibition with emapticap pegol (NOX-E36) in type 2 diabetic patients with albuminuria. Nephrol Dialys Transplant. (2016) 32:307–15. 10.1093/ndt/gfv459 PMC5410979 28186566

[254] Proudfoot AE Bonvin P Power CA. Targeting chemokines: pathogens can, why can't we? Cytokine. (2015). 74:259–67. 10.1016/j.cyto.2015.02.011 25753743

[255] Lukacs NW Oliveira SH Hogaboam CM Chemokines and asthma: redundancy of function or a coordinated effort? J Clin Invest. (1999) 104:995–9. 10.1172/JCI8125 10525033 PMC408861

[256] Power CA. Knock out models to dissect chemokine receptor function *in vivo*. J Immunol Methods. (2003) 273:73–82. 10.1016/S0022-1759(02)00419-2 12535799

[257] Solari R Pease JE Begg M. Chemokine receptors as therapeutic targets: why aren't there more drugs? Eur J Pharmacol. (2015) 746:363–7. 10.1016/j.ejphar.2014.06.060 25016087

[258] Schall TJ Proudfoot AE. Overcoming hurdles in developing successful drugs targeting chemokine receptors. Nat Rev Immunol. (2011) 11:355–63. 10.1038/nri2972 21494268

[259] Jacquelot N Duong C Belz G Zitvogel L. Targeting chemokines and chemokine receptors in melanoma and other cancers. Front Immunol. (2018) 9:2480. 10.3389/fimmu.2018.02480 30420855 PMC6215820

[260] Adage T Piccinini A-M Falsone A Trinker M Robinson J Gesslbauer B. Structure-based design of decoy chemokines as a way to explore the pharmacological potential of glycosaminoglycans. Br J Pharmacol. (2012) 167:1195–205. 10.1111/j.1476-5381.2012.02089.x 22747966 PMC3504987

[261] Triulzi T Forte L Regondi V Di Modica M Ghirelli C Carcangiu ML. HER2 signaling regulates the tumor immune microenvironment and trastuzumab efficacy. Oncoimmunology. (2019) 8:e1512942. 10.1080/2162402X.2018.1512942 30546951 PMC6287794

[262] Fader AN Rasool N Vaziri SA Kozuki T Faber PW Elson P. CCL2 expression in primary ovarian carcinoma is correlated with chemotherapy response and survival outcomes. Anticancer Res. (2010) 30:4791–8. 21187454

[263] Brault MS Kurt RA. Chemokines and antitumor immunity: walking the tightrope. Int Rev Immunol. (2003) 22:199–228. 10.1080/08830180305224 12745640

[264] Li M Knight DA Snyder LA Smyth MJ Stewart TJ. A role for CCL2 in both tumor progression and immunosurveillance. Oncoimmunology. (2013) 2:e25474. 10.4161/onci.25474 24073384 PMC3782157

